# Ischemic stroke: From pathological mechanisms to neuroprotective strategies

**DOI:** 10.3389/fneur.2022.1013083

**Published:** 2022-11-09

**Authors:** Yang Jiang, Zhenquan Liu, Yan Liao, Shuyong Sun, Yajie Dai, Yibo Tang

**Affiliations:** ^1^School of Traditional Chinese Medicine, Beijing University of Chinese Medicine, Beijing, China; ^2^School of Chinese Materia Medica, Beijing University of Chinese Medicine, Beijing, China

**Keywords:** ischemic stroke, inflammation, excitatory toxicity, oxidative stress, complement system, thrombus

## Abstract

Ischemic stroke (IS) has complex pathological mechanisms, and is extremely difficult to treat. At present, the treatment of IS is mainly based on intravenous thrombolysis and mechanical thrombectomy, but they are limited by a strict time window. In addition, after intravenous thrombolysis or mechanical thrombectomy, damaged neurons often fail to make ideal improvements due to microcirculation disorders. Therefore, finding suitable pathways and targets from the pathological mechanism is crucial for the development of neuroprotective agents against IS. With the hope of making contributions to the development of IS treatments, this review will introduce (1) how related targets are found in pathological mechanisms such as inflammation, excitotoxicity, oxidative stress, and complement system activation; and (2) the current status and challenges in drug development.

## Introduction

Ischemic stroke is one of the most common cerebrovascular diseases. With the aging of society, personal underlying diseases (such as hypertension, diabetes, heart disease, and hyperhomocysteinemia), smoking, alcohol consumption, and other factors, the incidence of IS has been continuously rising. According to the World Health Organization (WHO), more than 1.1 million people die each year from IS (1), showing that IS is seriously endangering people's health. The pathological mechanism of IS is very complex, including inflammation, excitotoxicity, oxidative stress, and the complement system, which eventually cause apoptosis and necrosis of neurons in the ischemic area. In the complex pathological mechanism of IS, inflammation was undervalued in the past because the brain was for a long time considered an immune-privileged organ, but its role is now more and more appreciated. Excitotoxicity is mainly caused by the increased glutamate and the subsequent calcium overload, which transformed the field of stroke research in the 1980s (2). Oxidative stress presents quite a challenge to ischemic tissue, particularly after reperfusion. Moreover, the activation of the complement system, thrombus formation, and pericyte death are important factors in triggering IS and subsequent neuronal death.

At present, intravenous thrombolysis and mechanical thrombectomy are the main treatment methods for IS, but they all have different restrictions. Intravenous thrombolytic therapy is usually represented by alteplase. Intravenous thrombolysis can be performed with alteplase within 4.5 h after an acute stroke, with the condition of excluding coagulation disorders and controlling blood pressure below 180/105 mmHg (3). The conventional regimen of clinical antiplatelet therapy is a combination of clopidogrel and aspirin. This dual antiplatelet treatment (a loading dose of 300 mg of clopidogrel plus 300 mg of aspirin, followed by a maintenance dose of 75 mg of clopidogrel and 75 mg of aspirin during the first 21 or 90 days), is effective in preventing IS after the onset of a transient ischemic attack (TIA) (4). Tenecteplase, a genetically modified variant of alteplase with increased fibrin specificity, shows similar safety and efficacy compared with alteplase in some clinical trials (5, 6). Compared to alteplase, tenecteplase has more advantages, such as a longer half-life, greater ease of use administered as a bolus medication, lower cost in some settings, and a higher incidence of reperfusion when combined with thrombectomy (6–8). These strengths may make it more promising in the treatment of IS. Mechanical thrombectomy, performed within 6 h after the onset of stroke, is another first-line treatment strategy for patients with ischemic stroke (3). However, mechanical thrombectomy is limited to the treatment of basilar artery occlusion in hospitals with related equipment and conditions (9).

In general, there are certain deficiencies in conventional treatment regimens, because of the strict time window, difficulty in delivering drugs to the central nervous system (CNS), and the inability to reverse the neuronal death that has already occurred. Even after the thrombus is removed, there is still a blockage of capillaries attributed to the dead pericytes losing their ability to regulate blood vessels in the brain (10). Furthermore, researchers need to pay more attention to ischemia-reperfusion injury in the brain after revascularization. Therefore, to overcome these difficulties and break these traditional constraints, researchers need to find potential targets and develop new neuroprotective agents for treatment strategies based on the pathological mechanism of IS. In this review, we mainly introduce the pathological mechanisms after IS such as inflammation, excitotoxicity, oxidative stress, complement system, and microcirculation.

## Inflammation

**Figure 1:**
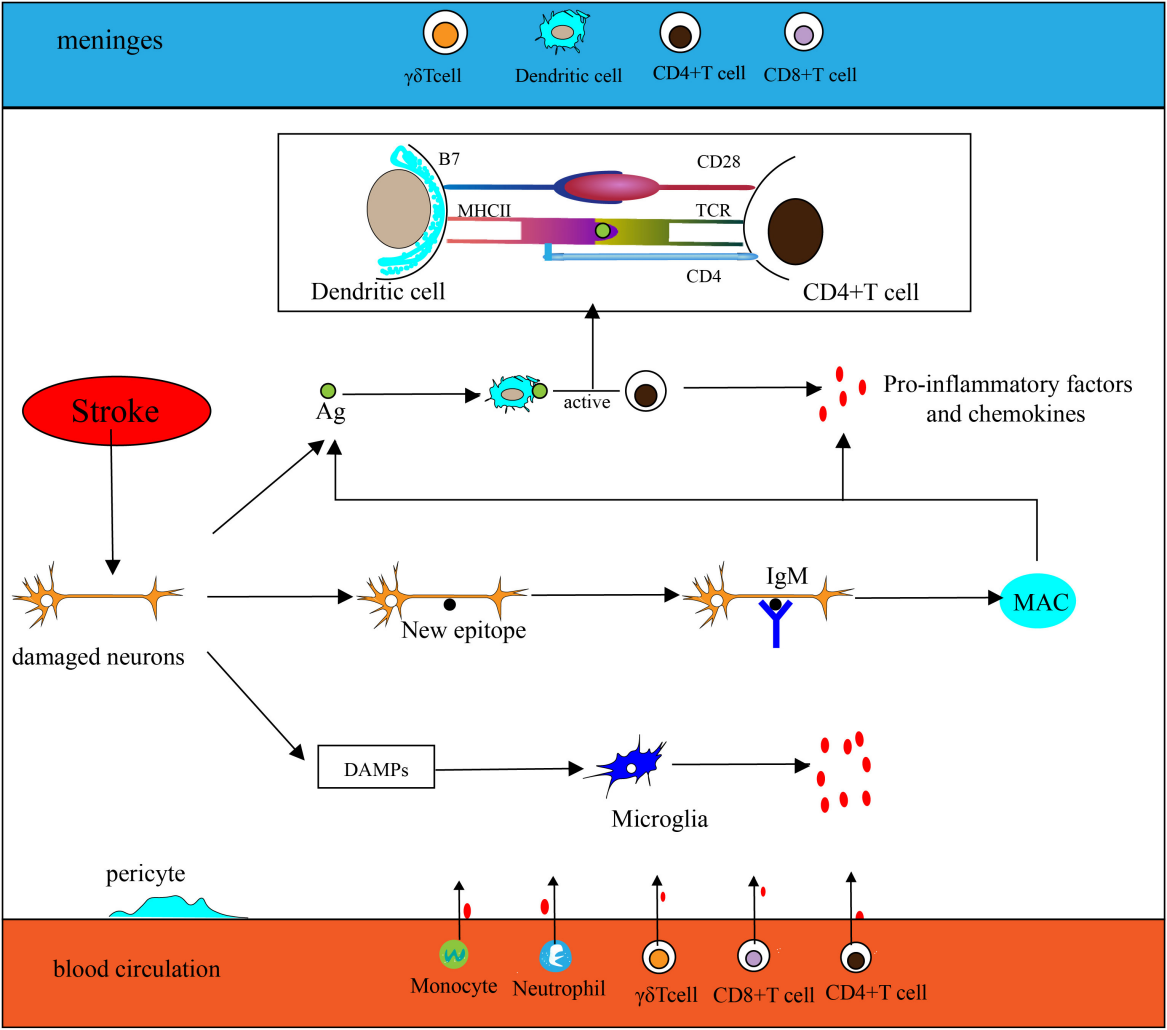
Inflammation in the ischemic brain. (1) Antigen (Ag) released by damaged neurons is ingested and processed by dendritic cells (DCs), then stimulates T cell activation (take CD4^+^ T cells as an example, CD4 T cells are activated by MHC II–peptide–TCR and CD28-B7). Activated T cells secrete pro-inflammatory factors and chemokines to participate in the inflammatory response. (2) Neurons are exposed to new epitopes after necrosis, which can be recognized by natural immunoglobulin IgM in brain tissue, thereby activating the classical pathway of the complement system and forming a membrane attack complex (MAC) eventually. Then cellular contents are released extracellularly by MAC, which exacerbates the inflammatory response. (3) Injured neurons release DAMPs to activate microglia and participate in the inflammatory response. (4) Chemokines secreted by microglia and other inflammatory cells promote peripheral phagocytes, neutrophils, CD4^+^T cells, CD8^+^T cells, and γδT cells to migrate toward CNS.

The extremely complex inflammatory response in the CNS consists of immune cells derived from the lymphatic circulation, resident microglia, monocytes, neutrophils that originate from the peripheral circulation, and cytokines secreted by various inflammatory cells after stroke. Innate immunity is rapidly involved in post-IS inflammation. Damage-associated molecular patterns (DAMPs) such as heat shock protein (HSP), high mobility group protein B1 (HMGB1), and hepatoma-derived growth factor (HDGF) can be recognized by pattern recognition receptors (PRRs) on some effector cells, thereby activating associated transcription factors and stimulating effector cells to secrete inflammatory factors. As part of the innate immune system, antigen-presenting cells (APCs) play a key role in the initiation of adaptive immunity. Dendritic cells (DCs) are one type of APC. Langerhans cells (a type of immature DCs) mature in lymphoid tissue after ingesting and processing autoantigen released by damaged tissue and necrotic neurons. Then DCs express peptide–MHC complexes along with highly expressed B7 (CD80/CD86), which generates a double stimulus for T cells, thereby mediating adaptive immunity. Inflammatory factors and chemokines are mainly produced by activated immune cells, inducing peripheral monocytes and neutrophils to migrate and infiltrate into the ischemic penumbra. In humoral immunity, B lymphocytes can differentiate into memory B cells and plasma cells that can produce antibodies. Although B cells may be involved in post-stroke pathologies, such as their ability to mediate delayed cognitive impairment following stroke (11), their effect on neuroinflammation is significantly weaker than that of T cells after acute ischemic stroke (AIS) (12). Furthermore, some experiments have shown that in the middle cerebral artery occlusion (MCAO) model, mice deficient in B lymphocytes exhibit no significant changes in cerebral infarct size and neural function compared with the wild-type (12), suggesting that B lymphocytes may not be the key to affecting neuroinflammation after acute stroke. Here, we mainly describe several inflammatory cells (Figure 1) that play important roles in the acute inflammatory response after stroke.

### Microglia

As a branch of the monocyte-phagocytic system, microglia are residents in the CNS, primarily responsible for immune surveillance and scavenging of pathogens and dying neurons (13). Microglia are the largest number of immune cells in the CNS, accounting for 5–10% of total brain cells (14). Microglia are involved in a variety of CNS diseases, including amyotrophic lateral sclerosis (ALS), IS, Alzheimer's disease (AD), meningeal inflammation, and schizophrenia (15–18). Microglia show contradictory functions in post-stroke inflammation (19). This may be due to their different phenotypes. Usually, microglia can be divided into three types: M0 (surveillance), M1 (pro-inflammatory), and M2 (anti-inflammatory) (19). M0 is primarily responsible for surveillance, with characteristics of low phagocytosis and inactivity (20, 21). In acute inflammation after stroke, M1 is generally considered to be activated earlier than M2. In fact, in the early stages of IS, the M2 type is the first to be activated, and its main function is to remove necrotic debris and protect brain tissue. Then M1 type mainly involved in brain tissue damage is activated (22). M1 type plays an important role in neuroinflammation after stroke, and the polarization of microglia to M1 has attracted a lot of attention. Many stimulatory factors cause the polarization of microglia toward M1, such as INF-γ secreted by Th1 cells activating the JAK/STAT pathway (22) or lipopolysaccharide stimulating Toll-Like receptor 4 (TLR4) on microglia (23). Activated M1 type can produce a variety of pro-inflammatory cytokines (IL-1β, TNF-α, IL-6, IL-23, IL-18, IL-12, CCL2, and CXCL10), reactive oxygen species (ROS), matrix metalloproteinase 9 (MMP9), and matrix metalloproteinase 3 (MMP3) leading to the apoptosis of neurons, the migrations of peripheral cells, the activation of immune cells, and the destruction of blood-brain barrier (BBB) (24–28). In contrast, M2 microglia mainly play an anti-inflammatory role and initiate neurogenesis, synaptogenesis, and neurovascular unit remodeling in the late stage of IS (29). In addition, the different polarization patterns of microglia may be related to the microenvironment, age, gender, temperature, and diabetes (30–34).

Promoting microglia polarization toward M2 while inhibiting M1 has emerged as a therapeutic approach for AIS. Minocycline (Table 1), as a selective inhibitor of M1 microglia, can be capable of reducing inflammation and promoting neurogenesis (35). In an open-label and evaluator-blinded study, patients with acute stroke had a significantly better outcome with minocycline treatment compared with placebo. This finding suggested a potential benefit of minocycline in AIS (36). Exendin-4 (Table 1), as a glucagon-like peptide receptor 1 agonist, in addition to its usage in blood glucose control, can promote the polarization of M2 microglia thereby providing neuroprotection and improving the prognosis of MCAO mice (37). In addition, two clinical trials on Exendin-4 treating IS are under recruitment. Hyperglycemia has been shown to activate M1 microglia (38), and the neuroprotective effects of Exendin-4 may be based on an indirect inhibition. Furthermore, the microenvironment exerting its influence on microglia is important for post-IS inflammation. For example, lacking folic acid (Table 1) will activate microglia *via* Notch/NF-kβ signaling in MCAO rats and BV-2 microglia after undergoing oxygen and glucose deprivation (OGD) (39). However, in two clinical trials of stroke, daily administration of folic acid, vitamin B6, and vitamin B12 did not seem to be more effective than a placebo in reducing the incidence of major vascular events, cognitive impairment, or cognitive decline (40, 41). In general, despite the difficulties in the transition to clinical, targeting microglia seems to be important in the treatment strategy of IS.

**Table 1:** Clinical trials and pre-clinical studies of drugs.

**Generic name**	**Target or signaling pathways**	**Pre-clinical references**	**Applications**	**Clinical trial**	**Phase**	**Results/status of the clinical trial**
**Anti-inflammatory**						
Natalizumab	Integrin α4β1	(158)	Mice	NCT02730455	2	Completed
Minocycline	microglia	(159)	Mice	NCT00930020	4	Terminated
Exendin-4	Microglia	(37)	Mice	NCT03287076	2	Active not recruiting
Folic acid/B12/B6	Microglia	(39)	Rat	NCT00354081	3	Completed
i6-FP	MAIT	(89)	Mice	—	—	—
Tak242	TLR4	(160)	Rat/*in vitro*	—	—	—
Isoquercetin	TLR4	(161)	Animals/*in vitro*	—	—	—
Dexmedetomidine	HMGB1/TLR4/NF-kB	(162)	Rat	NCT04916197	4	Recruiting
**Anti-excitotoxicity**						
NA-1	GluN2B-PSD95-NOS	(163)	Animals/neuronal	—	—	—
			Cultures			
ZL006	GluN2B-PSD95-NOS	(164)	Mice/rat	—	—	—
IC87201	GluN2B-PSD95-NOS	(165)	*In vitro*	—	—	—
β-lactam antibiotics	GLT-1	(103)	Mice/glial cell (gestation day 14–16 CD1 mice)	NCT05375240	2	Not yet recruiting
Memantine	Extrasynaptic NMDARs	(125, 126)	Rat/mice	NCT02535611/	3/0	Completed/active not recruiting
				NCT02144584		
Ketamine	NMDARs	(166)	Mice	NCT03223220	2, 3	Unknown
Geniposide	GluN2A/AKT/ERK	(167)	Rat	—	—	—
Pseudoginsenoside-F11	Akt-Creb	(168)	Rat		—	—
**Anti-oxidative stress**						
Acetylcysteine	TNF-α, iNOS, GSH, System x(c)-	(112, 113)	Rat	NCT04918719/	2/2	Not yet recruiting/
				NCT04920448		Not yet recruiting
Edaravone	ROS, RNS	(169)	Mice	NCT02430350	3	Completed
Uric acid	ROS	(170)	Mice	NCT00860366	2, 3	Completed
Melatonin	ROS, MDA, TNF-α	(171)	OGD/R-induced neuron	NCT05247125	4	Recruiting
tBHQ	Nrf2/ARE	(172)	Rat	—	—	—
Trans sodium crocetinate (TSC)	SIRT3/FOXO3a/SOD2	(173)	Rat	NCT03763929	2	Terminated
Genipin	UCP2-SIRT3	(174)	Mice	—	—	—
**Complement system**						
Human C1-esterase inhibitor	C1s, MASP-2	(157)	Mice	NCT01694381	0	Terminated
B4cry	IgM	(149, 150)	Mice	—	—	—
polyman2	MBL, C1s	(156)	Rats	—	—	—

### Macrophages

In rodents, according to the expression level of lymphocyte antigen 6 complex C1 (Ly6C) and chemokine receptors, monocytes are mainly divided into two subpopulations, namely pro-inflammatory subpopulations (Ly6C^high^CCR2^+^CX3CR1^low^) and anti-inflammatory subpopulations (Ly6C^low^CCR2^−^CX3CR1^high^) (42– 44). The CCL2-CCR2 axis is key to driving peripheral monocytes to the infarct area (45). Targeting the CCL2-CCR2 axis appears to be an ideal anti-inflammatory regimen due to the high expression of CCR2 in pro-inflammatory subpopulations. However, anti-inflammatory subsets in CNS are mainly transformed by infiltrating pro-inflammatory subsets rather than derived from peripheral monocytes (42), which makes targeting CCL2-CCR2 unfavorable to the prognosis of patients with IS from this perspective. Microglia were previously thought to be originated from peripheral macrophages because they have the same surface markers: CD11b, F4/80, and Iba-1 (46). In addition, microglia and macrophages have similar phenotypes, resulting in difficulty to distinguish them for researchers (47). Monocytes are now thought to be derived from hematopoietic stem cells (HSCs), whereas microglia are the descendants of yolk sac erythromyeloid progenitors (EMPs) (48). Researchers have found that, after IS, CXCR4 promotes monocyte infiltration and regional restriction of infarct tissue by macrophages derived from peripheral monocytes. Conversely, CXCR4 deficiency reduces the ability of monocytes to infiltrate the ischemic brain (49).

Macrophages are mainly derived from circulation, intestine, spleen, etc. In the early stage of IS, macrophages are induced to express triggering receptors expressed in myeloid cells 1 (TREM1), amplifying the inflammatory effects along with PRR (50). Interestingly, macrophages also contribute to neurogenesis. Mohle et al. believe that the reduction of neurons in the hippocampus is strongly correlated with the reduction of peripheral monocytes after oral antibiotics (51, 52). This suggests that macrophages may play a different role after a stroke. Macrophages derived from the spleen also get into CNS after IS. On the first day after cerebral infarction, the number of macrophages infiltrating CNS was significantly reduced in MCAO mice without spleen compared with the model group (53). Therefore, blocking the source of macrophages and preventing the differentiation of pro-inflammatory phenotype may be a strategy for the treatment of IS.

### Neutrophils

Neutrophils are the first leukocytes to infiltrate the ischemic brain after stroke, peaking at 48–72 h in CNS (54). The role of neutrophils in IS mainly includes the following aspects. The first role is secretory effect. There are many active substances such as MMP9, ROS, RNS, chemokines, and pro-inflammatory factors secreted by neutrophils, which mediate inflammation and the disruption of the BBB (55). Second, promoting thrombosis and blocking cerebrovascular, then leading to a no-reflow phenomenon after stroke (56). The third aspect is the neutrophil extracellular traps (NETs). The primary role of NETs is to capture and neutralize invading microorganisms (57), yet it is involved in the formation and stabilization of thrombus after stroke, which can lead to persistent ischemia in the brain (58). The fourth aspect is the phagocytosis of neutrophils. Neutrophils contribute to the clearance of necrotic tissue (59). The fifth aspect is the neurorestorative function. There is growing evidence that reparative neutrophil subsets and their products can be deployed to improve neurological outcomes (60, 61).

Similar to microglia and macrophages, conflicting phenotypes are also present in neutrophils. Neutrophils affected by tumor microenvironments can differentiate into two subtypes: N1 (anti-tumor) and N2 (pro-tumor) (62, 63). This contradictory phenotype also occurs in patients with stroke (55, 64). Targeting the neutrophil phenotype may also serve as an alternative to anti-neuroinflammation, and the ability of neutrophils to infiltrate the CNS also makes it a potential target for assisting drugs in getting into brain tissue (65).

### T cells

T cells develop, differentiate, and mature in the thymus, undergoing processes such as TCR development, positive selection, and negative selection. Then the vast majority of T cells transform into single-positive T cells (TCRαβ^+^CD4^+^CD8^−^ or TCRαβ^+^CD4^−^CD8^+^) mainly involved in adaptive immune, others mainly differentiate into TCRγδ^+^CD4^−^CD8^−^ T cells involved in innate immune. T cells in the spleen are involved in the pathological process of IS, resulting in reduced spleen volume and histomorphological changes (66). The involvement of T cells is often thought to aggravate brain damage but in some experiments the performance of T cells is contradictory. In mice with splenectomy, the neurological function is improved at the early stages of IS, but long-term neurological recovery is detrimental (67). In addition, studies have found that neurogenesis in the hippocampus is significantly reduced in mice lacking a complete immune system, especially those lacking CD4^+^T cells (68).

According to the leukocyte differentiation antigens, T cells are roughly divided into CD4^+^ T cells and CD8^+^ T cells, as well as mostly double-negative γδT (CD4^−^ CD8^−^) cells.

#### CD4^+^ T cells

CD4^+^T cells mainly include Th1, Th2, Th17, Th9, Th22, TFH, Treg, and other subpopulations. Th1 cells release the pro-inflammatory cytokines IFN-γ, IL-2, and TNF-α/β, and induce microglia and macrophage polarization toward M1 (69), which aggravates neuroinflammation after stroke. Contrary to Th1, Th2 promotes the polarization of microglia and macrophages toward the M2 type (70). Th1 and Th2 differ significantly in downstream cytokine lineages, and the Th1/Th2 mold can affect the outcomes of stroke (71). IL-33, as a member of the IL-1 family, improves MCAO mice's neurological deficit scores and reduces infarction volume by reducing IFN-γ^+^T cells and increasing Foxp3^+^ T cells in the spleen, thereby shifting Th1/Th2 mode to Th2 immune deviation and exerting a neuroprotective effect (70, 72). Similar to Th1, Th17 aggravates brain injury after a stroke. Intestinal Th17 are activated and then migrate into the meninges attributing to the CCL20-CCR6 axis after stroke (73). Furthermore, the CCR6-CCL20 axis can inhibit Treg differentiation and direct Tregs toward the pathogenic Th17-lineage (74). Targeting CCL20-CCR6 may be an ideal strategy for treating IS. Pioglitazone as a drug for the treatment of diabetes can reduce peripheral CCL20. Some animal experiments have shown that PG can reduce the inflammatory response after traumatic brain injury (TBI) (75), but whether PG can reduce neuroinflammation after stroke remains unclear. Treg is a special subset of Th2 that has been shown to negatively regulate neuroinflammation after stroke (76, 77). CD4^+^CD25^+^Foxp3^+^ Treg can inhibit neuroinflammation by producing the inhibitory cytokine TGF-β, IL-10, and IL-35 (78, 79). Treg cells have been a key topic in dealing with neuroinflammation after acute ischemic stroke in recent years.

#### CD8^+^ T cells

CD8^+^T cells are mainly cytotoxic T lymphocytes (CTL), which are the key to the occurrence of neuroinflammation after stroke. CTL mainly mediates cellular immunity and exerts cytotoxic effects on target cells. The function process of CTL is as follows: first, CTL cells bind target cells. CTL cells are activated by identifying the peptide-MHC-I complexes on target cells. Then the active substances in CTL are transferred to the immune synapse which is structured with a ternary structure (TCR–MHC–peptide) and surrounding adhesion molecules. Finally, CTL launches a lethal attack and mediates apoptosis through the perforin-granzyme pathway, Fas/Fasl pathway, and TNF-α pathway. Some studies have shown that consuming CD8^+^T cells show a better neuroprotective effect than consuming CD4^+^T cells, indicating that CD8^+^ T cells are more active than CD4^+^T cells in post-IS neuroinflammation (70, 80, 81). In addition, after peripheral CD8^+^ T cells were depleted, the infiltration of macrophages, neutrophils, and CD4^+^ T cells into the infarcted brain tissue in transient middle cerebral artery occlusion (TMCAO) mice was correspondingly reduced (82). The toxic effects of CD8^+^T cells can be achieved through the FASL-PDPK1 pathway and inhibition of PDPK1 can effectively improve neural function after stroke (83). Compared to CD4^+^T cells, targeted therapy for CD8^+^T cells may achieve a better outcome in acute IS.

#### Other T cells

After IS, non-specific immune T cells are also involved in the inflammatory response in the ischemic brain. γδT cells are distributed to the skin, intestines, airways, and other tissues after maturity in the thymus (84, 85), exerting an innate immune effect. Nasal-associated lymphoid tissue (NALT) may be one of the sources of γδT cells in the ischemic brain due to distance, but NALT ablation does not improve infarct size in stroke animals (84). After the proposal of the microbiome-gut-brain axis, γδT cells in the intestine are considered to be capable of migrating to the meninges after stroke, and the state of the microbiome in the gut can affect Treg/γδT cells ratio which is highly correlated with stroke outcomes (86). γδT cells predominantly secrete IL-17, mediating chronic inflammation after stroke, and promote the migration of neutrophils and monocytes to the ischemic brain, exacerbating stroke outcomes (86–88). Compared to other T cells, mucosal-associated invariant T (MAIT) cells were involved earlier in neuroinflammation after stroke. TMCAO mice with a MAIT deficiency or MAIT inhibitory ligand drugs (isobutyryl 6-formylpterin, i6-FP, in Table 1) showed a smaller infarct size compared to the model group (89). NKT cells are also part of non-specific immune T cells. Although NKT cells in cancer, hepatitis, pneumonia, and sepsis are increasingly valued (90–92), their involvement in neuroinflammation after IS requires further investigation.

Inflammation has been increasingly studied in IS since researchers moved away from the dogma that the brain is an immune-privileged organ. Additionally, this theory of immune privilege may rest on the low permeability of the BBB. However, the integrity of the BBB is disrupted after stroke, which facilitates the migration of peripheral inflammatory cells to the CNS (93, 94). In fact, a damaged BBB is not the only way inflammatory cells enter the ischemic brain. Currently, more investigators tend to support the theory that the choroid plexus is the main route of peripheral lymphocytes getting into the ischemic brain (95). Chemokines and chemokine receptors play a key role in the migration of inflammatory cells to the ischemic brain, such as CCR2-CCL2 related to most T cells (95), CCR6-CCL20 related to 17^+^ T cells (74), and CXCL1/CXCR2 related to neutrophils. But not all chemokines and chemokine receptors exacerbate neuroinflammation. For example, the CXCR3/CXCL10 axis, which served as the brain-homing mechanism for CD8^+^CD122^+^CD49d^lo^ T regulatory-like cells, can provide neuroprotection in MCAO mice (96). Therefore, regulating peripheral inflammatory cells or regulatory-like cell migration could serve as a strategy for treating IS. In addition, it is important to regulate the activation of inflammatory cells in the CNS. Although the emergence of the brain-gut axis theory may make the gut microbiota and pathogen-associated molecular patterns (PAMPs), the initiators of neuroinflammation, sterile inflammation triggered by DAMPs remain the main type after the onset of IS. DAMPs internalization was largely mediated by the class A scavenger receptors MSR1 that was regulated by the transcription factor Mafb (97). MSR1 and Mafb may be promising targets for treating IS. Finally, targeting inflammatory cells themselves or the inflammatory factors they produce can provide enlightenment for future drug development for IS.

## Excitatory toxicity

Excitatory toxicity is mainly caused by the increased glutamate (Glu) in the ischemic brain, which leads to neuronal necrosis and apoptosis by a series of biochemical cascades. After an acute stroke, mitochondrial ATP production decreases due to cerebral ischemia and hypoxia. Then intracellular and extracellular ion disorders (intracellular: Na^+^, Ca^2+^, Cl^−^ increase; extracellular: K^+^ increase) caused by the dysfunctional ATP-dependent ion pump will eventually lead to an increase in glutamate in the extracellular or synaptic cleft. For example, intracellular transport of glutamate dependent on a normal Na^+^ gradient (extracellular Na^+^ are more than intracellular) is regulated by an ATP-dependent Na^+^ pump. The normal Na^+^ gradient is reversed (intracellular Na^+^ are more than extracellular) when ATP synthesis is reduced and the ATP-dependent Na^+^ pump is deactivated, which ultimately increases glutamate in the extracellular or synaptic cleft. As glutamate-mediated excitotoxicity severely affects prognosis in patients with stroke, studying the production and metabolism of glutamate and the downstream pathways mediated by glutamate receptors have great potential for the development of neuroprotective drugs against IS.

### Production and metabolism of glutamate

**Figure 2:**
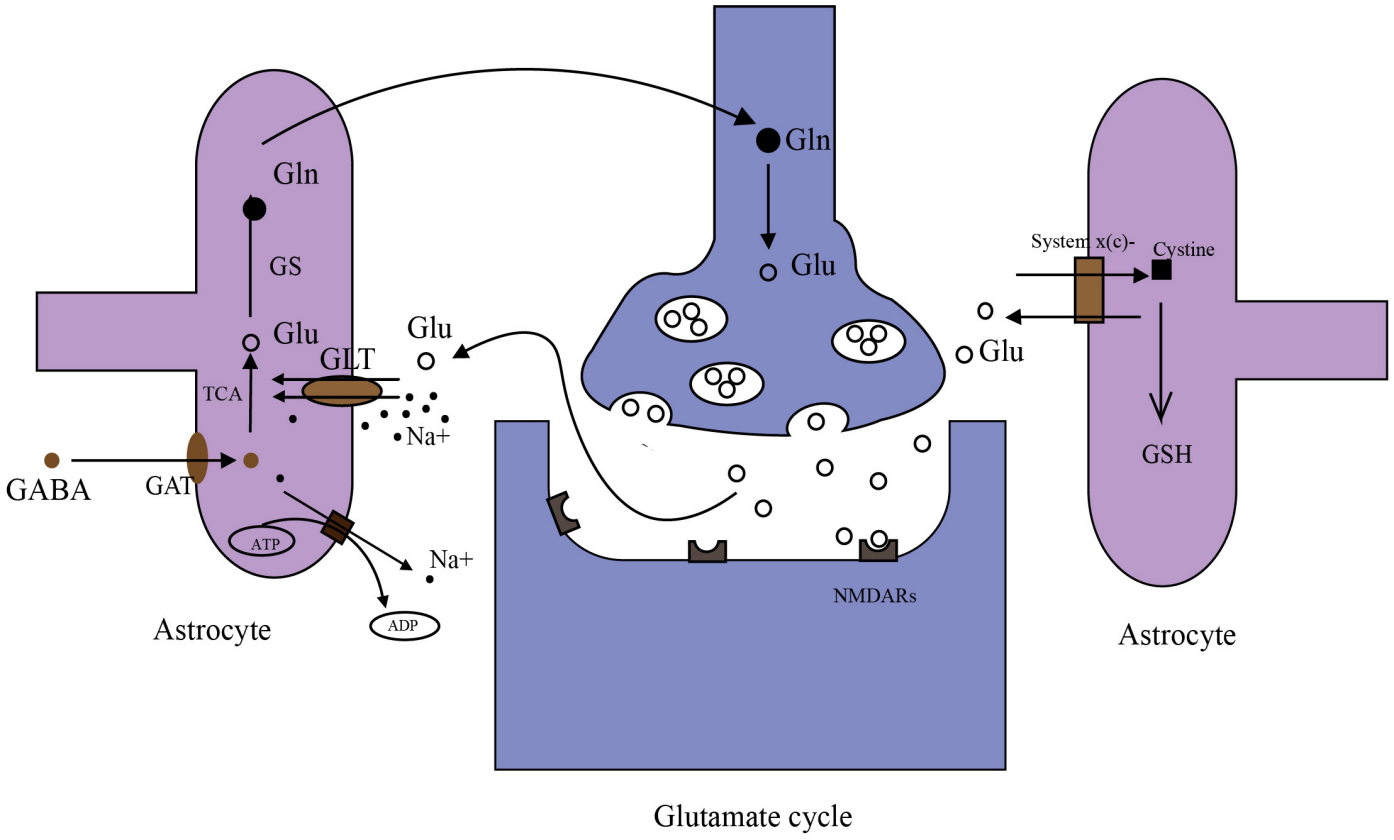
Metabolism of glutamate in the brain. (1) Glutamate (Glu)–glutamine (Gln) cycle: when nerve impulses are transmitted to the postsynaptic membrane, the neurotransmitter glutamate (Glu) stored in synaptic vesicles is released into the synaptic cleft and binds with glutamate receptors (NMDARs). Glutamate transporters (GLT-1 shown in the figure) on astrocyte transport Glu together with Na^+^ into astrocytes. Na^+^ in astrocytes is transferred to extracellular by an ATP-dependent Na^+^ pump. Glu in astrocytes is converted to Gln by GS, then Gln is taken up by synapses and converted to Glu in synapses. Glu is stored in synaptic vesicles again. (2) γ aminobutyric acid (GABA) is taken up by GABA transporters (GATs) in astrocytes. Then GABA can be converted to Glu through the tricarboxylic acid cycle (TAC). (3) System x(c)- transports cystine into cells in exchange for the Glu. Cystine synthesizes glutathione (GSH) in astrocytes.

Glutamate in the brain originates from multiple pathways (Figure 2). Glutamate in the periphery does not enter the brain under physiological conditions due to BBB. However, studies have shown that glutamate levels in the brain can be reduced by peritoneal dialysis after stroke (98). The main reasons for this phenomenon may be as follows. First, disrupted BBB. After the stroke, a high concentration of glutamate in brain tissue and a low concentration of glutamate in blood will form a gradient (99, 100). Disrupted BBB may facilitate glutamate to enter the periphery. Second, glutamate in the brain generates glutamine, which can directly cross BBB into the periphery. Then peripheral glutamate regenerated by glutamine is cleared by peritoneal dialysis. There is a glutamate-glutamine cycle in neurons and their neighboring astrocytes (Figure 2) (101, 102) and many targets in this cycle are important for extracellular glutamate production. Glutamate transporter GLT-1 is a member of excitatory amino acid transporters (EAATs), and it is capable of removing glutamate from the synaptic cleft. β-lactam antibiotics (Table 1) can stimulate the activity of GLT-1 in mice glial cells after OGD (103). Glutamine synthase (GS) is the speed limit of the glutamate-glutamine cycle (104) and can be degraded by reactive oxygen species (ROS) after stroke, resulting in the accumulation of glutamate (105). Targeting GS may be an ideal strategy for treating IS.

The glutamate/cystine antiporter system x(c)- transports cystine into cells in exchange for neurotransmitter glutamate at a ratio of 1:1 (106, 107). Cystine ingested into the cell produces cysteine, which is used as a raw material for the synthesis of glutathione (GSH) and participates in the scavenging of intracellular free radicals (106, 107). System x(c)- relies on a gradient of glutamate, and when extracellular glutamate concentration increases, the transfer of cystine into cells decreases, resulting in oxidative free radical damage (107–109). Acetylcysteine (Table 1) contributes to the scavenging of this oxidative free radical by stimulating glutamate/cystine antiporter system x(c) and promoting the generation of GSH (110, 111). Acetylcysteine protects against injury in a rat model of focal cerebral ischemia and ischemia/reperfusion, respectively (112, 113). In addition, two clinical trials registered with acetylcysteine treating IS, are still ongoing (114).

### Glutamate and glutamate receptors

Glutamate exerts excitotoxic effects through glutamate receptors (NMDARs). NMDARs have a dual role in neurons, which may depend on the subtype of NMDARs (115). NMDARs consist of two NR1 subunits and one other subunit (NR2A/B/C/D or NR3A/B). NMDARs containing NR2A subunits mainly promote neuronal survival, while NMDARs containing NR2B subunits mainly mediate neuronal excitotoxicity and promote apoptosis (116). Another argument for the dual role of NMDARs has to do with their location. Studies have shown that there are two distinct subtypes of NMDARs: extrasynaptic NMDARs and intrasynaptic NMDARs (117). Stimulation of intrasynaptic NMDARs can activate CREBs in a variety of ways (115, 118). Activated CREB can enhance mitochondrial tolerance to cellular stress (119) and inhibit the pro-death transcription factor by promoting the expression of brain-derived neurotrophic factor (BDNF) (115), exerting an anti-apoptotic effect. Conversely, stimulating extrasynaptic NMDARs can dephosphorylate CREBs by inhibiting Ros/ERK1/2 pathway, which activates pro-apoptotic genes in the bcl-2 family, and thus induces apoptosis (120, 121).

In short, extrasynaptic NMDARs and intrasynaptic NMDARs play opposite roles. Various subtypes have different affinities with glutamate, and their positional relationships can both serve as explanations for glutamate excitotoxicity. In other words, physiological glutamate levels fail to activate extrasynaptic NMDARs (low affinity or low level of glutamate), but they can activate intrasynaptic NMDARs (high affinity or high level of glutamate) exerting a pro-survival signal. Extrasynaptic NMDARs can only be activated when the glutamate concentration rises above a certain threshold to exert a pro-death signal. Blocking NMDARs has been a potential target for inhibiting glutamate excitatory toxicity, particularly blocking extrasynaptic NMDARs and NMDARs containing NR2B subunit. Compared with intrasynaptic NMDARs, memantine (Table 1) can inhibit extrasynaptic receptors more effectively (122), and it has been clinically used in the treatment of AD in the US. However, memantine only shows efficacy in patients with severe and moderate AD (123). For mild AD, a meta-analysis found no difference between memantine and placebo in cognition, activities of daily living, or behavior (124). In a preclinical study of IS, memantine blunted the noxious effects of delayed thrombolysis on lesion volumes and neurological deficits in MCAO mice (125) and exerted synergistic neuroprotective effects with clenbuterol in MCAO rats (126). However, *in vivo* experiment of Trotman et al., higher doses of memantine (20 mg/kg/day) significantly increased injury. Similar results were also found in their *in vitro* experiments. Therefore, a proper dosage of memantine is significant in future clinical trials (127). Ifenprodil is capable of binding to NMDARs containing NR2B subunit with a high affinity (128, 129). Although clinical trials of ifenprodil are currently only conducted in idiopathic pulmonary fibrosis (IPF)/corona virus disease 2019 (COVID-19)/post-traumatic stress disorder (PTSD), using ifenprodil in treatment against IS remains promising.

### Glutamate toxicity

When glutamate binds with the receptor, an action potential is formed. After excitotoxicity occurs, glutamate continues to excite receptors and keeps Na^+^ channels open. Then intracellular osmotic pressure increases due to this persistently opened Na^+^ channel, resulting in acute neuronal death. When neurons are in a resting state, Ca^2+^ channels are blocked by Mg^2+^. When glutamate binds to NMDARs, Mg^2+^ is removed and Ca^2+^ channels are opened by the depolarized postsynaptic membrane. Intracellular Ca^2+^ is elevated by these opened Ca^2+^ channels, which contributes to the activation of a ternary structure (PSD95-NR2B-NOS) (130). The activated ternary structure then releases nitric oxide synthase (NOS) leading to the production of reactive nitrogen species (RNS). Cell *in vitro* experiments and animal experiments have demonstrated that disrupting this ternary structure can reduce glutamate-mediated excitotoxicity and improve neuronal tolerance to glutamate (131, 132). Furthermore, mitochondria are disturbed by calcium overload to release a large number of oxidative free radicals, which can promote neuronal apoptosis and aggravate calcium overload again through activated transient receptor potential melastatin-subfamily member 7 (TRPM7) and transient receptor potential melastatin-subfamily member 2 (TRPM2) (115). Recently, Zong et al. discovered that TRPM2 directly interacts with GluN2a/b of extrasynaptic NMDARs through the unique EE3 motif in its N-tail and the KKR motifs in the C-tail of GluN2a/b. This coupling mechanism plays an important role in the excitotoxicity of ischemic brain injury in mice (133). Therefore, in downstream of glutamate-mediated excitotoxicity, some ion channels that mediate calcium overload, some complexes that mediate oxidative stress, and the coupling of TRPM2 and extrasynaptic NMDARs are expected to be targets for the treatment of IS.

NMDAR-mediated excitotoxicity has been extensively studied in stroke; however, NMDAR antagonists face challenges now in the treatment of ischemic stroke in human patients. This may be attributed to some key targets and structures that have not been fully studied. Additionally, we hope to provide some inspiration for future drug development through the above summary of excitotoxicity.

## Oxidative stress

### Generation of oxidative free radicals

**Figure 3:**
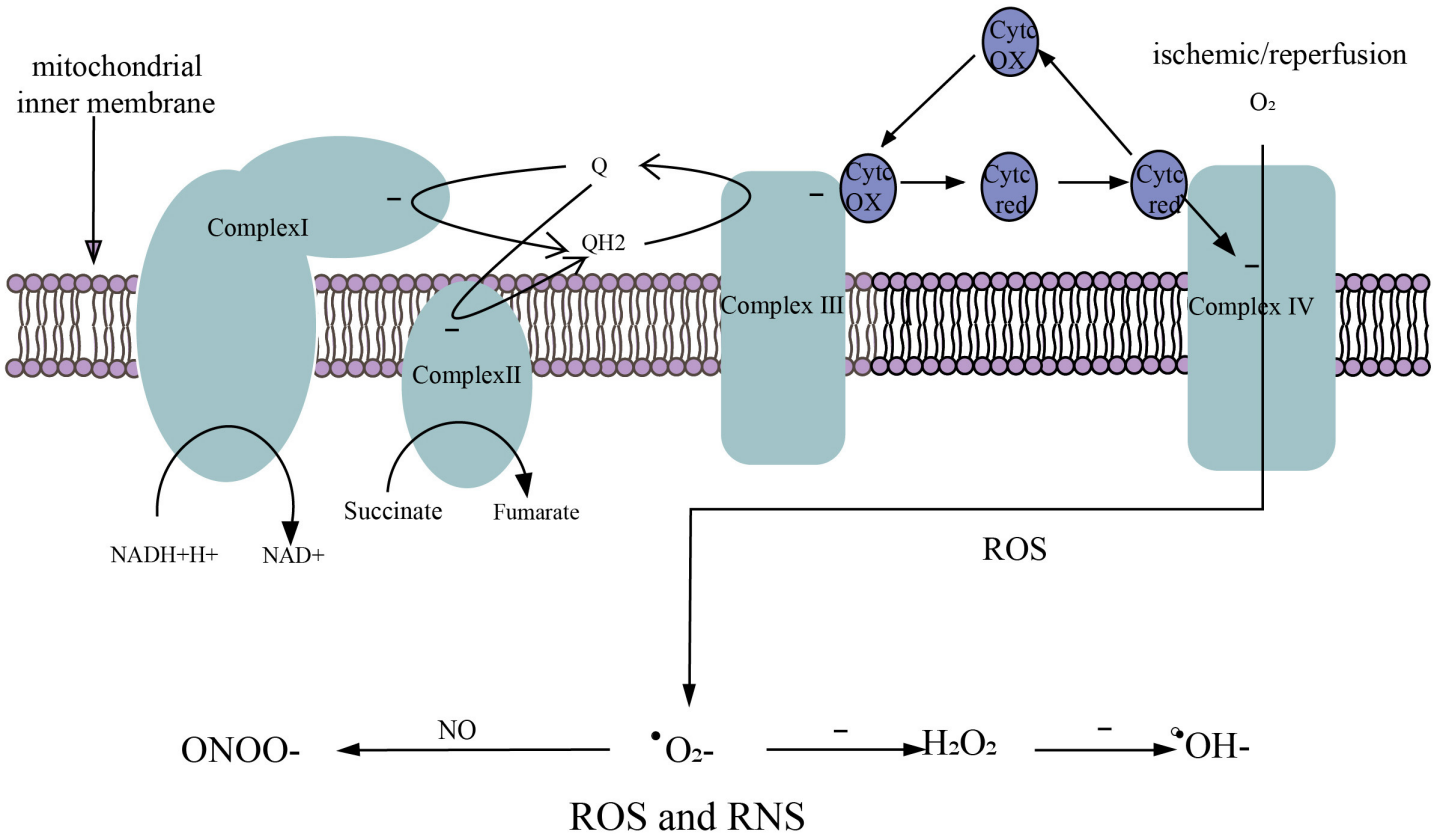
Generation of ROS and RNS. (1) NADH oxidation respiratory chain. NADH-H+ transfers electrons to complex I and is oxidized to NAD^+^. (2) Succinate oxidation respiratory chain. Succinate transfers electrons to complex II and is oxidized to fumarate. (3) The electrons in complex I/II are captured by ubiquinone (Q) to form QH2. The electrons in QH2 are transferred to complex III, then QH2 that loses electrons becomes Q to participate in the electron transfer again. Electrons in complex III are transferred to complex IV *via* cytochrome c (Cytc). (4) O_2_ obtains electrons from complex IV to generate ROS. (5) After ischemic/reperfusion injury, O_2_ gets a single electron to generate superoxide anion (^**.**^${\text{O}}_{2}^{-}$), ^**.**^${\text{O}}_{2}^{-}$ accepts a single electron to generate hydrogen peroxide (H_2_O_2_), and H_2_O_2_ accepts a single electron to be reduced to hydroxyl radical (^**.**^OH^−^), ^**.**^${\text{O}}_{2}^{-}$ can rapidly oxidize NO to produce nitrite (ONOO^−^).

Physiologically, there is stable oxidation and antioxidant system in the body. In the process of reperfusion of the ischemic brain, glucose, and oxygen enter the brain again. Oxidative glycolysis of glucose produces a large amount of reduced NADH-H+ and FADH2 along with superoxide anion generated in the process of electron transfer, resulting in excessive ROS and RNS, as well as damage to the ischemic brain (134). It is currently believed that there are two oxidative respiratory chains (Figure 3) in mitochondria, one is NADH oxidation respiratory chain (NADH-complex I-CoQ-complex III-Cytc-complex IV-O_2_) and the other is the FADH2 oxidation respiratory chain, also called succinate oxidation respiratory chain (succinate -complex II-CoQ-complex III-Cytc-complex IV-O2). Studies have shown that FADH2 accumulation can be attributed to the reversal of succinate dehydrogenase (SDH) after stroke. In the early stage of perfusion, the accumulated FADH2 activates SDH and drives mitochondria to produce a large amount of ROS *via* reverse electron transfer (RET) (105). Excessive ROS and RNS promote lipid peroxidation, mitochondrial and DNA damage, protein nitrification and oxidation, and depletion of antioxidants (135). In addition to this, ROS and RNS lead to the overexpression of inflammatory genes, inflammatory and chemokine production, BBB disruption, leukocyte recruitment, and cerebral edema (136).

Removing ROS and RNS is a major strategy to treat IS and protect neurons. Edaravone (Table 1) is clinically used for the treatment of IS, which can scavenge oxidative free radicals and achieve the purpose of protecting ischemic neurons. Uric acid (Table 1) contributes to the scavenging of ROS, including nitrite, which can reduce cerebral infarct volume after stroke and improve neurological outcomes after transient or permanent cerebral ischemia in rodents (137–139).

### Antioxidant enzyme system

There are various antioxidant enzymes and small molecule antioxidants (such as vitamin C/E, ubiquinone, β-carotene) in our body, which together constitute the antioxidant system. Superoxide dismutase (SOD) is widely distributed in the human body, responsible for catalyzing.O_2_- and converting it to oxygen and hydrogen peroxide (H_2_O_2_), and it is an important part of the cellular antioxidant system (140). Catalase (CAT) has a strong catalytic ability to H_2_O_2_. However, CAT will inevitably generate.OH- in the process of scavenging H_2_O_2_. Tri-manganese (III) salen-based cryptands, an analog of CAT, can minimize the production of.OH- while removing H_2_O_2_ (141). Glutathione peroxidase (GPx) is the main enzyme in the body to scavenge ROS, removing H_2_O_2_ and other peroxides. Furthermore, GPx is the key to inhibiting neuronal ferroptosis (142). A selenocysteine-containing peptide, Tat SelPep, can increase GPx expression by binding to nuclear DNA and effectively improve stroke outcomes (143). Thioredoxin (Trx), also one of the body's antioxidant enzymes, has been found to improve outcomes after stroke. Melatonin (MT), a neurohormone in the human body, can achieve anti-oxidation by regulating Trx (144). However, the performance of most antioxidant drugs in animal experiments is not satisfactory, which may be related to the inability of antioxidants to target mitochondria, the birthplace of ROS and RNS (145). Therefore, this kind of antioxidant drug targeting mitochondria needs to be further considered in the future.

Scavenging oxidative free radicals and increasing the reserves of antioxidant enzymes have been one of the strategies for the treatment of IS. However, only edaravone is currently approved for the clinical treatment of IS. Additional mechanisms related to oxidative stress help resolve this dilemma. For example, ferroptosis is a new form of cell death caused by an increase in iron ion-dependent lipid peroxides (146). Although the specific mechanism of ferroptosis in IS has not been elucidated, inhibition of lipid peroxidation and regulation of iron metabolism is promising for treating IS.

## Complement system

The complement system is composed of complement intrinsic components, complement regulatory proteins, and complement receptors, and its activation pathways are mainly three-fold: classical pathway (CP), bypass pathway (AP), and lectin pathway (LP). The complement system is part of innate immunity and is involved in the subsequent phases of humoral immunity. Since B lymphocytes are barely detectable in brain tissue within a week after stroke (11, 12), CP that rely on immune complexes (ICs) for activation may be limited in the acute phase of IS. However, there is a natural immunoglobulin M (IgM) in the brain, which can recognize a new epitope on damaged neurons and still activate CP to exert pathological damage after stroke (147, 148). The endpoint of the complement cascade is the formation of a membrane attack complex (MAC), resulting in cell rupture and death. In recent years, neuroprotective agents targeting C3 and C5 or complement fragments have entered our field of vision. As a fusion construct, B4cry inhibits IgM binding to the new epitope, C3 cleavage, and the activation of microglia, which can reduce complement deposition in ischemic lesions and improve neurological function after stroke (149, 150). C5a-C5AaR axis capable of promoting neutrophil migration (151) is widely involved in COVID-19-related coagulopathy, viral hepatitis, cancer, and myocarditis (152–155), and this axis may serve as a target for the treatment of IS. Mannan-binding lectin (MBL) plays an important role in the activation of LP. Using polyman2 (the synthesized mannosylated molecule selected for its binding to MBL) or anti-MBL antibody, can inhibit the activation of mannan-binding lectin-associated serine protease-2 (MASP-2) to block the subsequent complement cascade, and exert a neuroprotective effect on TMCAO or permanent middle cerebral artery occlusion (PMCAO) rats (156). Complement regulatory proteins are one of the long-term targets that complement drug developers are focusing on. C1 inhibitor (C1-INH) capable of inhibiting C1s or MASP-2 can inhibit complement cascade from the upstream of C3. Animal experiments have shown that compared with TMCAO mice, C1-INH-deficient TMCAO mice showed larger ischemic foci and worse neurobehavioral performance (157).

There are many components in the complement system, but there is little research on the complement system in IS. Since the development of neuroprotective agents against IS has mostly failed, the complement system seems to be a good path.

## Thrombus and pericytes

Thrombus is the fundamental factor causing cerebral vascular obstruction and cerebral ischemia and hypoxia. For thrombus formation, the importance of the involvement of ultra-large (UL) von Willebrand factor (ULVWF) and collagen–von Willebrand factor–glycoprotein Ib axis has been highlighted in many studies (175, 176). ULVWF multimers are regulated by a metalloproteinase Adamts13. Some studies have shown that low activity of Adamts13 is associated with an increased risk of IS and TIA (177). The interaction of platelets and neutrophils is critical in thrombosis. Platelets promote the formation of neutrophil extracellular traps (NETs) (178), which can be cleared by patients' deoxyribonuclease-1 (DNase-1) in stroke treatment (179). Antithrombotic therapy has always been the main method for IS treatment. However, some limitations of thrombolysis, such as the therapeutic window and low efficacy of reperfusion, are difficult to solve. In recent years, the combination of thrombolysis and neuroprotection seems to be an excellent strategy for treating IS. For example, uric acid helps to scavenge ROS. In a pre-clinical study by Romanos et al. (138), uric acid and recombinant tissue plasminogen activator (rtPA) showed a synergistic effect in the model of thromboembolic cerebral ischemia in rats. A clinical trial (NCT00860366) on the combination of rtPA and uric acid in the treatment of IS has been completed. Unfortunately, we have not found relevant research results. In addition, in a double-blind, placebo-controlled, phase 2b trial, the combination of rtPA and uric acid may prevent early ischemic deterioration after acute stroke in patients with thrombolysis (180). This strategy of thrombolysis combined with neuroprotection can be a good point in the treatment of IS in the future.

In addition to being interrupted by a thrombus, cerebral blood flow is also regulated by capillary pericytes. Ischemic pericytes constrict and compress capillaries after stroke, reducing cerebral blood flow. More importantly, pericyte death attributed to sustained ischemia and hypoxia can lead to irreversible constriction of capillaries and permanent interruption of blood flow (10). The time of pericyte resistance to ischemia and hypoxia may affect the time window of intravenous thrombolysis or mechanical thrombectomy. Preventing the shrinkage and death of pericytes and improving the tolerance of pericytes to ischemia and hypoxia may be of great significance to prolong this time window.

## How to deliver drugs to the CNS?

We have described many promising drugs earlier, but all of these drugs have to face a common problem: It is difficult to deliver them to the CNS. The BBB is the main barrier preventing drugs from entering the CNS. It has been reported that <2% of small molecule drugs with CNS affects approved by the Food and Drug Administration (FDA) can pass through the intact BBB (181, 182). The existence of the BBB is necessary for maintaining the homeostasis of the cerebral microenvironment and ensuring the normal functions of the CNS. Nevertheless, the BBB also impedes the intracerebral delivery of therapeutic agents (183). Although studies have shown that the occurrence of IS can destroy and increase the permeability of the BBB, the BBB remains the main obstacle for drugs to overcome (184).

At present, scientists and industry have developed a variety of technologies to deliver drugs to the CNS. For instance, the emergence of nanoparticles provides a new strategy for drugs to enter the CNS. Nanoparticles have been proven to deliver a great variety of drugs across the BBB, and this mechanism of crossing BBB now appears to be receptor-mediated endocytosis of the brain capillary endothelial cells, followed by transcytosis (185, 186). By combining with different drugs, nanoparticles perform three major approaches for ischemic stroke therapy: recanalization, neuroprotection, and combination therapy (184). More importantly, nanoparticles are capable of increasing drug bioavailability, enhancing therapeutic efficacy, and reducing unwanted toxicity (184). In recent years, exosomes have been mentioned as a strategy for the treatment of IS. Exosomes are endosome-derived membrane-bound vesicles with diameters of 30–150 nm, and they are released by most cell types (187–189). Among the cargoes carried by exosomes, miRNAs are valued by researchers because they may be the core of the therapeutic effects of exosomes (189). Therefore, how to select miRNA contained in exosomes may become a strategy for treating IS. Additionally, in the treatment of IS, engineered exosomes that contain selected miRNA have been proven to be more effective compared with naïve exosomes (189). Although the ability of exosomes to directly cross the BBB is uncertain, several studies have shown that some exosomes could cross the BBB in healthy and inflamed brains (189–191).

In addition to the aforementioned nanoparticles and exosomes, neurotropic virus mediation emerged as a strategy for the treatment of IS. Neurotropic viruses, with an affinity for nerve, can cross the BBB through multiple pathways, such as direct transcytosis, virus-infected immune cells, and retrograde transport from peripheral nerves to the CNS (192). Additionally, this property makes neurotropic viruses a CNS-targeting strategy. Carrier-mediated transcytosis (CMT) and receptor-mediated transcytosis (RMT) are long-term concerns of researchers. However, when delivering drugs to the CNS through RMT or CMT, the target receptor or carrier protein should be highly expressed in the endothelial cells of the cerebral vasculature, especially those in the microvascular (192). Therefore, this carrier protein that can match the receptors abundantly expressed in cerebral microvascular needs to be elaborately designed.

Crossing the BBB seems to be an inescapable obstacle for delivering drugs to the CNS, even though there are now many ways to bypass the BBB, such as highly invasive intracerebral injection, intranasal, retro-orbital, or intrathecal administration. However, these methods of bypassing the BBB are difficult to achieve clinically, which may be attributed to their operational complexity, high invasiveness, and low bioavailability. With the development of technology, RMT-based strategies, neurotropic virus-based approaches, nanoparticles, exosomes, etc. have shown great potential to deliver drugs to the CNS.

## Difficulties in clinical transformation

At present, most neuroprotective drugs are facing difficulties in clinical transformation. These difficulties are caused by many aspects. Animal models of IS, where middle cerebral arteries are often blocked by nylon fibers, have been questioned because they do not reflect the occurrence of vascular embolism under natural conditions (193). Moreover, there are many differences between animal models and humans, such as age, species, and underlying diseases. The side effects of drugs are also important factors. For example, in the development of complement drugs, complement inhibitors inevitably inhibit the activity of serum complement while inhibiting complement activation, which increases the risk of infection (194). Therefore, it is necessary to develop more targeted drugs that have higher precision. More importantly, incomplete mechanism research will also lead to failure in drug development. For example, although the use of anti-IL-17A drugs has seen efficacy in the treatment of psoriasis, it is clinically invalid in the treatment of amyotrophic lateral sclerosis (ALS), rheumatoid disease, and experimental autoimmune encephalomyelitis (EAE) (195). This contradictory role in different diseases may be attributed to the incompleteness of the mechanism such as the possible duality of IL-17, the different pathogenicities of Th17, and the negative feedback of IL-17 (195, 196). In addition, the ability of the drug to penetrate the BBB, oral bioavailability, half-life, and statistical bias are also factors that determine whether the drug can be successfully translated into the clinic.

## Conclusion

Since most clinical patients with ischemic stroke fail in conventional treatments such as intravenous thrombolysis and mechanical thrombectomy due to missed time windows, it is of great significance to find other strategies to protect CNS. In the complex pathological mechanisms of IS, we can obtain many methods and targets for the treatment of IS. It is very promising to develop new drugs for IS from these mechanisms and targets. Additionally, combining these new drugs with brain delivery technologies and more precise targeted therapy may go further in the clinical treatment of IS.

## Author contributions

YJ and YT conceived the topic and determined the outline of this review. YJ, YT, and ZL contributed to the manuscript writing. YD and SS collected the literature and finished the figures and tables. YT, ZL, and YL critically revised the manuscript. All authors contributed to the article and approved the submitted version.

## Funding

Key project at the central government level: The ability establishment of sustainable use for valuable Chinese medicine resources (2060302).

## Conflict of interest

The authors declare that the research was conducted in the absence of any commercial or financial relationships that could be construed as a potential conflict of interest.

## Publisher's note

All claims expressed in this article are solely those of the authors and do not necessarily represent those of their affiliated organizations, or those of the publisher, the editors and the reviewers. Any product that may be evaluated in this article, or claim that may be made by its manufacturer, is not guaranteed or endorsed by the publisher.

## References

[1] Chen Z Jiang B Ru X Sun H Sun D Liu X. Mortality of stroke and its subtypes in china: results from a nationwide population-based survey. Neuroepidemiology. (2017) 48:95–102. 10.1159/000477494 28586776

[2] Chamorro A Dirnagl U Urra X Planas AM. Neuroprotection in acute stroke: targeting excitotoxicity, oxidative and nitrosative stress, and inflammation. Lancet Neurol. (2016) 15:869–81. 10.1016/S1474-4422(16)00114-9 27180033

[3] Powers WJ. Acute ischemic stroke. N Engl J Med. (2020) 383:252–60. 10.1056/NEJMcp1917030 32668115

[4] Amarenco P. Transient ischemic attack. Reply N Engl J Med. (2020) 383:1598. 10.1056/NEJMc2022610 33053300

[5] Logallo N Novotny V Assmus J Kvistad CE Alteheld L Ronning OM. Tenecteplase versus alteplase for management of acute ischaemic stroke (NOR-TEST): a phase 3, randomised, open-label, blinded endpoint trial. Lancet Neurol. (2017) 16:781–8. 10.1016/S1474-4422(17)30253-3 28780236

[6] Menon BK Buck BH Singh N Deschaintre Y Almekhlafi MA Coutts SB. Intravenous tenecteplase compared with alteplase for acute ischaemic stroke in Canada (AcT): a pragmatic, multicentre, open-label, registry-linked, randomised, controlled, non-inferiority trial. Lancet. (2022) 400:161–9. 35779553 10.1016/S0140-6736(22)01054-6

[7] Campbell BCV Mitchell PJ Churilov L Yassi N Kleinig TJ Dowling RJ. Tenecteplase versus alteplase before thrombectomy for ischemic stroke. N Engl J Med. (2018) 378:1573–82. 29694815 10.1056/NEJMoa1716405

[8] Patel P Yavagal D Khandelwal P. Hyperacute management of ischemic strokes: JACC focus seminar. J Am Coll Cardiol. (2020) 75:1844–56. 10.1016/j.jacc.2020.03.006 32299596

[9] Gerschenfeld G Muresan IP Blanc R Obadia M Abrivard M Piotin M. Two paradigms for endovascular thrombectomy after intravenous thrombolysis for acute ischemic stroke. JAMA Neurol. (2017) 74:549–56. 10.1001/jamaneurol.2016.5823 28319240 PMC5822198

[10] Hall CN Reynell C Gesslein B Hamilton NB Mishra A Sutherland BA. Capillary pericytes regulate cerebral blood flow in health and disease. Nature. (2014) 508:55–60. 10.1038/nature13165 24670647 PMC3976267

[11] Doyle KP Quach LN Sole M Axtell RC Nguyen TV Soler-Llavina GJ. B-lymphocyte-mediated delayed cognitive impairment following stroke. J Neurosci. (2015) 35:2133–45. 10.1523/JNEUROSCI.4098-14.2015 25653369 PMC4315838

[12] Kleinschnitz C Schwab N Kraft P Hagedorn I Dreykluft A Schwarz T. Early detrimental T-cell effects in experimental cerebral ischemia are neither related to adaptive immunity nor thrombus formation. Blood. (2010) 115:3835–42. 10.1182/blood-2009-10-249078 20215643

[13] Colonna M Butovsky O. Microglia function in the central nervous system during health and neurodegeneration. Annu Rev Immunol. (2017) 35:441–68. 10.1146/annurev-immunol-051116-052358 28226226 PMC8167938

[14] Thion MS Ginhoux F Garel S. Microglia and early brain development: an intimate journey. Science. (2018) 362:185–9. 10.1126/science.aat0474 30309946

[15] Xiong X Gu L Wang Y Luo Y Zhang H Lee J. Glycyrrhizin protects against focal cerebral ischemia *via* inhibition of T cell activity and HMGB1-mediated mechanisms. J Neuroinflammation. (2016) 13:241. 10.1186/s12974-016-0705-5 27609334 PMC5016958

[16] Manberg A Skene N Sanders F Trusohamn M Remnestal J Szczepinska A. Altered perivascular fibroblast activity precedes ALS disease onset. Nat Med. (2021) 27:640–6. 10.1038/s41591-021-01295-9 33859435 PMC7613336

[17] Yilmaz M Yalcin E Presumey J Aw E Ma M Whelan CW. Overexpression of schizophrenia susceptibility factor human complement C4A promotes excessive synaptic loss and behavioral changes in mice. Nat Neurosci. (2021) 24:214–24. 10.1038/s41593-020-00763-8 33353966 PMC8086435

[18] Wood H. Microglial changes associated with meningeal inflammation in multiple sclerosis. Nat Rev Neurol. (2021) 17:262. 10.1038/s41582-021-00494-9 33846617

[19] Franco R Fernandez-Suarez D. Alternatively activated microglia and macrophages in the central nervous system. Prog Neurobiol. (2015) 131:65–86. 10.1016/j.pneurobio.2015.05.003 26067058

[20] Kettenmann H Hanisch UK Noda M Verkhratsky A. Physiology of microglia. Physiol Rev. (2011) 91:461–553. 10.1152/physrev.00011.2010 21527731

[21] Eldahshan W Fagan SC Ergul A. Inflammation within the neurovascular unit: focus on microglia for stroke injury and recovery. Pharmacol Res. (2019) 147:104349. 10.1016/j.phrs.2019.104349 31315064 PMC6954670

[22] Hu X Li P Guo Y Wang H Leak RK Chen S. Microglia/macrophage polarization dynamics reveal novel mechanism of injury expansion after focal cerebral ischemia. Stroke. (2012) 43:3063–70. 10.1161/STROKEAHA.112.659656 22933588

[23] Kigerl KA Gensel JC Ankeny DP Alexander JK Donnelly DJ Popovich PG. Identification of two distinct macrophage subsets with divergent effects causing either neurotoxicity or regeneration in the injured mouse spinal cord. J Neurosci. (2009) 29:13435–44. 10.1523/JNEUROSCI.3257-09.2009 19864556 PMC2788152

[24] Ito S Kimura K Haneda M Ishida Y Sawada M Isobe K. Induction of matrix metalloproteinases (MMP3, MMP12 and MMP13) expression in the microglia by amyloid-beta stimulation via the PI3K/Akt pathway. Exp Gerontol. (2007) 42:532–7. 10.1016/j.exger.2006.11.012 17198748

[25] Toyama T Hoshi T Noguchi T Saito Y Matsuzawa A Naganuma A. Methylmercury induces neuronal cell death by inducing TNF-alpha expression through the ASK1/p38 signaling pathway in microglia. Sci Rep. (2021) 11:9832. 10.1038/s41598-021-89210-7 33972601 PMC8110582

[26] Zhao Y Wei ZZ Lee JH Gu X Sun J Dix TA. Pharmacological hypothermia induced neurovascular protection after severe stroke of transient middle cerebral artery occlusion in mice. Exp Neurol. (2020) 325:113133. 10.1016/j.expneurol.2019.113133 31770520

[27] Boche D Perry VH Nicoll JA. Review: activation patterns of microglia and their identification in the human brain. Neuropathol Appl Neurobiol. (2013) 39:3–18. 10.1111/nan.12011 23252647

[28] Hu X Zhang M Leak RK Gan Y Li P Gao Y. Delivery of neurotherapeutics across the blood brain barrier in stroke. Curr Pharm Des. (2012) 18:3704–20. 10.2174/138161212802002715 22574984

[29] Wang X Xuan W Zhu ZY Li Y Zhu H Zhu L. The evolving role of neuro-immune interaction in brain repair after cerebral ischemic stroke. CNS Neurosci Ther. (2018) 24:1100–14. 10.1111/cns.13077 30350341 PMC6489764

[30] Nishimoto R Derouiche S Eto K Deveci A Kashio M Kimori Y. Thermosensitive TRPV4 channels mediate temperature-dependent microglia movement. Proc Natl Acad Sci U S A. (2021) 118:e2012894118. 10.1073/pnas.2012894118 33888579 PMC8092382

[31] Han J Fan Y Zhou K Blomgren K Harris RA. Uncovering sex differences of rodent microglia. J Neuroinflammation. (2021) 18:74. 10.1186/s12974-021-02124-z 33731174 PMC7972194

[32] Ma S Wang J Wang Y Dai X Xu F Gao X. Diabetes mellitus impairs white matter repair and long-term functional deficits after cerebral ischemia. Stroke. (2018) 49:2453–63. 10.1161/STROKEAHA.118.021452 30355111 PMC6205761

[33] Jackson-Cowan L Eldahshan W Dumanli S Dong G Jamil S Abdul Y. Delayed administration of angiotensin receptor (AT2R) agonist C21 improves survival and preserves sensorimotor outcomes in female diabetic rats post-stroke through modulation of microglial activation. Int J Mol Sci. (2021) 22:1356. 10.3390/ijms22031356 33572986 PMC7866408

[34] Olah M Patrick E Villani AC Xu J White CC Ryan KJ. A transcriptomic atlas of aged human microglia. Nat Commun. (2018) 9:539. 10.1038/s41467-018-02926-5 29416036 PMC5803269

[35] Kobayashi K Imagama S Ohgomori T Hirano K Uchimura K Sakamoto K. Minocycline selectively inhibits M1 polarization of microglia. Cell Death Dis. (2013) 4:e525. 10.1038/cddis.2013.54 23470532 PMC3613832

[36] Lampl Y Boaz M Gilad R Lorberboym M Dabby R Rapoport A. Minocycline treatment in acute stroke: an open-label, evaluator-blinded study. Neurology. (2007) 69:1404–10. 10.1212/01.wnl.0000277487.04281.db 17909152

[37] Darsalia V Hua S Larsson M Mallard C Nathanson D Nystrom T. Exendin-4 reduces ischemic brain injury in normal and aged type 2 diabetic mice and promotes microglial M2 polarization. PLoS ONE. (2014) 9:e103114. 10.1371/journal.pone.0103114 25101679 PMC4125154

[38] Dong LD Ma YM Xu J Guo YZ Yang L Guo FY. Effect of hyperglycemia on microglial polarization after cerebral ischemia-reperfusion injury in rats. Life Sci. (2021) 279:119660. 10.1016/j.lfs.2021.119660 34052292

[39] Cheng M Yang L Dong Z Wang M Sun Y Liu H. Folic acid deficiency enhanced microglial immune response *via* the Notch1/nuclear factor kappa B p65 pathway in hippocampus following rat brain I/R injury and BV2 cells. J Cell Mol Med. (2019) 23:4795–807. 10.1111/jcmm.14368 31087489 PMC6584545

[40] Group VTS. B vitamins in patients with recent transient ischaemic attack or stroke in the VITAmins TO Prevent Stroke (VITATOPS) trial: a randomised, double-blind, parallel, placebo-controlled trial. Lancet Neurol. (2010) 9:855–65. 10.1016/S1474-4422(10)70187-3 20688574

[41] Hankey GJ Ford AH Yi Q Eikelboom JW Lees KR Chen C. Effect of B vitamins and lowering homocysteine on cognitive impairment in patients with previous stroke or transient ischemic attack: a prespecified secondary analysis of a randomized, placebo-controlled trial and meta-analysis. Stroke. (2013) 44:2232–9. 10.1161/STROKEAHA.113.001886 23765945

[42] Han D Liu H Gao Y. The role of peripheral monocytes and macrophages in ischemic stroke. Neurol Sci. (2020) 41:3589–607. 10.1007/s10072-020-04777-9 33009963

[43] Li Q Barres BA. Microglia and macrophages in brain homeostasis and disease. Nat Rev Immunol. (2018) 18:225–42. 10.1038/nri.2017.125 29151590

[44] Auffray C Fogg D Garfa M Elain G Join-Lambert O Kayal S. Monitoring of blood vessels and tissues by a population of monocytes with patrolling behavior. Science. (2007) 317:666–70. 10.1126/science.1142883 17673663

[45] Huang Y Wang J Cai J Qiu Y Zheng H Lai X. Targeted homing of CCR2-overexpressing mesenchymal stromal cells to ischemic brain enhances post-stroke recovery partially through PRDX4-mediated blood-brain barrier preservation. Theranostics. (2018) 8:5929–44. 10.7150/thno.28029 30613272 PMC6299433

[46] Ginhoux F Greter M Leboeuf M Nandi S See P Gokhan S. Fate mapping analysis reveals that adult microglia derive from primitive macrophages. Science. (2010) 330:841–5. 10.1126/science.1194637 20966214 PMC3719181

[47] Yamasaki R Lu H Butovsky O Ohno N Rietsch AM Cialic R. Differential roles of microglia and monocytes in the inflamed central nervous system. J Exp Med. (2014) 211:1533–49. 10.1084/jem.20132477 25002752 PMC4113947

[48] Gomez Perdiguero E Klapproth K Schulz C Busch K Azzoni E Crozet L. Tissue-resident macrophages originate from yolk-sac-derived erythro-myeloid progenitors. Nature. (2015) 518:547–51. 10.1038/nature13989 25470051 PMC5997177

[49] Werner Y Mass E Ashok Kumar P Ulas T Handler K Horne A. Cxcr4 distinguishes HSC-derived monocytes from microglia and reveals monocyte immune responses to experimental stroke. Nat Neurosci. (2020) 23:351–62. 10.1038/s41593-020-0585-y 32042176 PMC7523735

[50] Liu Q Johnson EM Lam RK Wang Q Bo Ye H Wilson EN. Peripheral TREM1 responses to brain and intestinal immunogens amplify stroke severity. Nat Immunol. (2019) 20:1023–34. 10.1038/s41590-019-0421-2 31263278 PMC6778967

[51] Baruch K Schwartz M. Circulating monocytes in between the gut and the mind. Cell Stem Cell. (2016) 18:689–91. 10.1016/j.stem.2016.05.008 27257756

[52] Mohle L Mattei D Heimesaat MM Bereswill S Fischer A Alutis M. Ly6C(hi) monocytes provide a link between antibiotic-induced changes in gut microbiota and adult hippocampal neurogenesis. Cell Rep. (2016) 15:1945–56. 10.1016/j.celrep.2016.04.074 27210745

[53] Kim E Yang J Beltran CD Cho S. Role of spleen-derived monocytes/macrophages in acute ischemic brain injury. J Cereb Blood Flow Metab. (2014) 34:1411–9. 10.1038/jcbfm.2014.101 24865998 PMC4126087

[54] Jickling GC Liu D Ander BP Stamova B Zhan X Sharp FR. Targeting neutrophils in ischemic stroke: translational insights from experimental studies. J Cereb Blood Flow Metab. (2015) 35:888–901. 10.1038/jcbfm.2015.45 25806703 PMC4640255

[55] Garcia-Culebras A Duran-Laforet V Pena-Martinez C Ballesteros I Pradillo JM Diaz-Guzman J. Myeloid cells as therapeutic targets in neuroinflammation after stroke: specific roles of neutrophils and neutrophil-platelet interactions. J Cereb Blood Flow Metab. (2018) 38:2150–64. 10.1177/0271678X18795789 30129391 PMC6282223

[56] Ames A 3rd Wright RL Kowada M Thurston JM Majno G. Cerebral ischemia. II. The no-reflow phenomenon. Am J Pathol. (1968) 52:437–53. 5635861 PMC2013326

[57] Marin-Esteban V Turbica I Dufour G Semiramoth N Gleizes A Gorges R. Afa/Dr diffusely adhering *Escherichia coli* strain C1845 induces neutrophil extracellular traps that kill bacteria and damage human enterocyte-like cells. Infect Immun. (2012) 80:1891–9. 10.1128/IAI.00050-12 22371374 PMC3347451

[58] Genchi A Semerano A Gullotta GS Strambo D Schwarz G Bergamaschi A. Cerebral thrombi of cardioembolic etiology have an increased content of neutrophil extracellular traps. J Neurol Sci. (2021) 423:117355. 10.1016/j.jns.2021.117355 33647733

[59] Roberts RE Elumalai GL Hallett MB. Phagocytosis and motility in human neutrophils is competent but compromised by pharmacological inhibition of ezrin phosphorylation. Curr Mol Pharmacol. (2018) 11:305–15. 10.2174/1874467211666180516100613 29766831

[60] Sas AR Carbajal KS Jerome AD Menon R Yoon C Kalinski AL. A new neutrophil subset promotes CNS neuron survival and axon regeneration. Nat Immunol. (2020) 21:1496–505. 10.1038/s41590-020-00813-0 33106668 PMC7677206

[61] Bird L. Neurorestorative neutrophils. Nat Rev Immunol. (2021) 21:2–3. 10.1038/s41577-020-00485-9 33257820

[62] Sionov RV Fridlender ZG Granot Z. The multifaceted roles neutrophils play in the tumor microenvironment. Cancer Microenviron. (2015) 8:125–58. 10.1007/s12307-014-0147-5 24895166 PMC4714999

[63] Mantovani A. The yin-yang of tumor-associated neutrophils. Cancer Cell. (2009) 16:173–4. 10.1016/j.ccr.2009.08.014 19732714

[64] Cuartero MI Ballesteros I Moraga A Nombela F Vivancos J Hamilton JA. N2 neutrophils, novel players in brain inflammation after stroke: modulation by the PPARgamma agonist rosiglitazone. Stroke. (2013) 44:3498–508. 10.1161/STROKEAHA.113.002470 24135932

[65] Zhang C Ling CL Pang L Wang Q Liu JX Wang BS. Direct macromolecular drug delivery to cerebral ischemia area using neutrophil-mediated nanoparticles. Theranostics. (2017) 7:3260–75. 10.7150/thno.19979 28900508 PMC5595130

[66] Seifert HA Hall AA Chapman CB Collier LA Willing AE Pennypacker KR. transient decrease in spleen size following stroke corresponds to splenocyte release into systemic circulation. J Neuroimmune Pharmacol. (2012) 7:1017–24. 10.1007/s11481-012-9406-8 23054371 PMC3518577

[67] Ran Y Liu Z Huang S Shen J Li F Zhang W. Splenectomy fails to provide long-term protection against ischemic stroke. Aging Dis. (2018) 9:467–79. 10.14336/AD.2018.0130 29896434 PMC5988601

[68] Wolf SA Steiner B Akpinarli A Kammertoens T Nassenstein C Braun A. CD4-positive T lymphocytes provide a neuroimmunological link in the control of adult hippocampal neurogenesis. J Immunol. (2009) 182:3979–84. 10.4049/jimmunol.0801218 19299695

[69] Durafourt BA Moore CS Zammit DA Johnson TA Zaguia F Guiot MC. Comparison of polarization properties of human adult microglia and blood-derived macrophages. Glia. (2012) 60:717–27. 10.1002/glia.22298 22290798

[70] Wang S Zhang H Xu Y. Crosstalk between microglia and T cells contributes to brain damage and recovery after ischemic stroke. Neurol Res. (2016) 38:495–503. 10.1080/01616412.2016.1188473 27244271

[71] Wong CH Jenne CN Tam PP Leger C Venegas A Ryckborst K. Prolonged activation of invariant natural killer T cells and TH2-skewed immunity in stroke patients. Front Neurol. (2017) 8:6. 10.3389/fneur.2017.00006 28154551 PMC5244395

[72] Xiao W Guo S Chen L Luo Y. The role of Interleukin-33 in the modulation of splenic T-cell immune responses after experimental ischemic stroke. J Neuroimmunol. (2019) 333:576970. 10.1016/j.jneuroim.2019.576970 31146104

[73] Reboldi A Coisne C Baumjohann D Benvenuto F Bottinelli D Lira S. C-C chemokine receptor 6-regulated entry of TH-17 cells into the CNS through the choroid plexus is required for the initiation of EAE. Nat Immunol. (2009) 10:514–23. 10.1038/ni.1716 19305396

[74] Kulkarni N Meitei HT Sonar SA Sharma PK Mujeeb VR Srivastava S. CCR6 signaling inhibits suppressor function of induced-Treg during gut inflammation. J Autoimmun. (2018) 88:121–30. 10.1016/j.jaut.2017.10.013 29126851

[75] Das M Tang X Han JY Mayilsamy K Foran E Biswal MR. CCL20-CCR6 axis modulated traumatic brain injury-induced visual pathologies. J Neuroinflammation. (2019) 16:115. 10.1186/s12974-019-1499-z 31151410 PMC6544928

[76] Rodriguez-Perea AL Gutierrez-Vargas J Cardona-Gomez GP Guarin CJ Rojas M Hernandez PA. Atorvastatin modulates regulatory T cells and attenuates cerebral damage in a model of transient middle cerebral artery occlusion in rats. J Neuroimmune Pharmacol. (2017) 12:152–62. 10.1007/s11481-016-9706-5 27614888

[77] Liesz A Suri-Payer E Veltkamp C Doerr H Sommer C Rivest S. Regulatory T cells are key cerebroprotective immunomodulators in acute experimental stroke. Nat Med. (2009) 15:192–9. 10.1038/nm.1927 19169263

[78] Li P Gan Y Sun BL Zhang F Lu B Gao Y. Adoptive regulatory T-cell therapy protects against cerebral ischemia. Ann Neurol. (2013) 74:458–71. 10.1002/ana.23815 23674483 PMC3748165

[79] Wan YY. Multi-tasking of helper T cells. Immunology. (2010) 130:166–71. 10.1111/j.1365-2567.2010.03289.x 20557575 PMC2878461

[80] Yilmaz G Granger DN. Leukocyte recruitment and ischemic brain injury. Neuromolecular Med. (2010) 12:193–204. 10.1007/s12017-009-8074-1 19579016 PMC2878882

[81] Yilmaz G Arumugam TV Stokes KY Granger DN. Role of T lymphocytes and interferon-gamma in ischemic stroke. Circulation. (2006) 113:2105–12. 10.1161/CIRCULATIONAHA.105.593046 16636173

[82] Selvaraj UM Ujas TA Kong X Kumar A Plautz EJ Zhang S. Delayed diapedesis of CD8 T cells contributes to long-term pathology after ischemic stroke in male mice. Brain Behav Immun. (2021) 95:502–13. 10.1016/j.bbi.2021.05.001 33964435 PMC8221572

[83] Fan L Zhang CJ Zhu L Chen J Zhang Z Liu P. FasL-PDPK1 pathway promotes the cytotoxicity of CD8(+) T cells during ischemic stroke. Transl Stroke Res. (2020) 11:747–61. 10.1007/s12975-019-00749-0 32036560

[84] Brea D Poon C Murphy M Lubitz G Iadecola C Anrather J. Ablation of nasal-associated lymphoid tissue does not affect focal ischemic brain injury in mice. PLoS ONE. (2018) 13:e0205470. 10.1371/journal.pone.0205470 30300386 PMC6177188

[85] Prinz I Silva-Santos B Pennington DJ. Functional development of gammadelta T cells. Eur J Immunol. (2013) 43:1988–94. 10.1002/eji.201343759 23928962

[86] Benakis C Brea D Caballero S Faraco G Moore J Murphy M. Commensal microbiota affects ischemic stroke outcome by regulating intestinal gammadelta T cells. Nat Med. (2016) 22:516–23. 10.1038/nm.4068 27019327 PMC4860105

[87] Shichita T Sugiyama Y Ooboshi H Sugimori H Nakagawa R Takada I. Pivotal role of cerebral interleukin-17-producing gammadeltaT cells in the delayed phase of ischemic brain injury. Nat Med. (2009) 15:946–50. 10.1038/nm.1999 19648929

[88] Gelderblom M Weymar A Bernreuther C Velden J Arunachalam P Steinbach K. Neutralization of the IL-17 axis diminishes neutrophil invasion and protects from ischemic stroke. Blood. (2012) 120:3793–802. 10.1182/blood-2012-02-412726 22976954

[89] Nakajima S Tanaka R Yamashiro K Chiba A Noto D Inaba T. Mucosal-associated invariant T cells are involved in acute ischemic stroke by regulating neuroinflammation. J Am Heart Assoc. (2021) 10:e018803. 10.1161/JAHA.120.018803 33733818 PMC8174378

[90] Kreutmair S Unger S Nunez NG Ingelfinger F Alberti C De Feo D. Distinct immunological signatures discriminate severe COVID-19 from non-SARS-CoV-2-driven critical pneumonia. Immunity. (2021) 54:1578–93.e5. 10.1016/j.immuni.2021.05.002 34051147 PMC8106882

[91] Ma C Han M Heinrich B Fu Q Zhang Q Sandhu M. Gut microbiome-mediated bile acid metabolism regulates liver cancer *via* NKT cells. Science. (2018) 360:eaan5931. 10.1126/science.aan5931 29798856 PMC6407885

[92] Yan J Mitra A Hu J Cutrera JJ Xia X Doetschman T. Corrigendum to “Interleukin-30 (IL27p28) alleviates experimental sepsis by modulating cytokine profile in NKT cells”. J Hepatol. (2016) 64:1128–36. 10.1016/j.jhep.2016.04.028 26767500 PMC4834232

[93] Amoo M O'Halloran PJ Henry J Husien MB Brennan P Campbell M. Permeability of the blood-brain barrier after traumatic brain injury; radiological considerations. J Neurotrauma. (2021) 39:20–34. 10.1089/neu.2020.7545 33632026

[94] Gwak MG Chang SY. Gut-brain connection: microbiome, gut barrier, and environmental sensors. Immune Netw. (2021) 21:e20. 10.4110/in.2021.21.e20 34277110 PMC8263213

[95] Llovera G Benakis C Enzmann G Cai R Arzberger T Ghasemigharagoz A. The choroid plexus is a key cerebral invasion route for T cells after stroke. Acta Neuropathol. (2017) 134:851–68. 10.1007/s00401-017-1758-y 28762187

[96] Cai W Shi L Zhao J Xu F Dufort C Ye Q. Neuroprotection against ischemic stroke requires a specific class of early responder T cells in mice. J Clin Invest. (2022) 132:e157678. 10.1172/JCI157678 35912857 PMC9337834

[97] Shichita T Ito M Morita R Komai K Noguchi Y Ooboshi H. MAFB prevents excess inflammation after ischemic stroke by accelerating clearance of damage signals through MSR1. Nat Med. (2017) 23:723–32. 10.1038/nm.4312 28394332

[98] Godino Mdel C Romera VG Sanchez-Tomero JA Pacheco J Canals S Lerma J. Amelioration of ischemic brain damage by peritoneal dialysis. J Clin Invest. (2013) 123:4359–63. 10.1172/JCI67284 23999426 PMC3784528

[99] Zaghmi A Dopico-Lopez A Perez-Mato M Iglesias-Rey R Hervella P Greschner AA. Sustained blood glutamate scavenging enhances protection in ischemic stroke. Commun Biol. (2020) 3:729. 10.1038/s42003-020-01406-1 33273696 PMC7713697

[100] da Silva-Candal A Perez-Diaz A Santamaria M Correa-Paz C Rodriguez-Yanez M Arda A. Clinical validation of blood/brain glutamate grabbing in acute ischemic stroke. Ann Neurol. (2018) 84:260–73. 10.1002/ana.25286 30014516

[101] Sonnewald U Qu H Aschner M. Pharmacology and toxicology of astrocyte-neuron glutamate transport and cycling. J Pharmacol Exp Ther. (2002) 301:1–6. 10.1124/jpet.301.1.1 11907150

[102] Nagy Z Nardai S. Cerebral ischemia/repefusion injury: from bench space to bedside. Brain Res Bull. (2017) 134:30–7. 10.1016/j.brainresbull.2017.06.011 28625785

[103] Rothstein JD Patel S Regan MR Haenggeli C Huang YH Bergles DE. Beta-lactam antibiotics offer neuroprotection by increasing glutamate transporter expression. Nature. (2005) 433:73–7. 10.1038/nature03180 15635412

[104] Mostafeezur RM Shinoda M Unno S Zakir HM Takatsuji H Takahashi K. Involvement of astroglial glutamate-glutamine shuttle in modulation of the jaw-opening reflex following infraorbital nerve injury. Eur J Neurosci. (2014) 39:2050–9. 10.1111/ejn.12562 24666367

[105] Chouchani ET Pell VR Gaude E Aksentijevic D Sundier SY Robb EL. Ischaemic accumulation of succinate controls reperfusion injury through mitochondrial ROS. Nature. (2014) 515:431–5. 10.1038/nature13909 25383517 PMC4255242

[106] Dringen R Hirrlinger J. Glutathione pathways in the brain. Biol Chem. (2003) 384:505–16. 10.1515/BC.2003.059 12751781

[107] Albrecht P Lewerenz J Dittmer S Noack R Maher P Methner A. Mechanisms of oxidative glutamate toxicity: the glutamate/cystine antiporter system xc- as a neuroprotective drug target. CNS Neurol Disord Drug Targets. (2010) 9:373–82. 10.2174/187152710791292567 20053169

[108] Phillis JW Ren J O'Regan MH. Transporter reversal as a mechanism of glutamate release from the ischemic rat cerebral cortex: studies with DL-threo-beta-benzyloxyaspartate. Brain Res. (2000) 868:105–12. 10.1016/S0006-8993(00)02303-9 10841893

[109] Griffith OW. Biologic and pharmacologic regulation of mammalian glutathione synthesis. Free Radic Biol Med. (1999) 27:922–35. 10.1016/S0891-5849(99)00176-8 10569625

[110] Conrad M Sato H. The oxidative stress-inducible cystine/glutamate antiporter, system x (c) (-) : cystine supplier and beyond. Amino Acids. (2012) 42:231–46. 10.1007/s00726-011-0867-5 21409388

[111] Moss HG Brown TR Wiest DB Jenkins DD. N-Acetylcysteine rapidly replenishes central nervous system glutathione measured *via* magnetic resonance spectroscopy in human neonates with hypoxic-ischemic encephalopathy. J Cereb Blood Flow Metab. (2018) 38:950–8. 10.1177/0271678X18765828 29561203 PMC5999009

[112] Sekhon B Sekhon C Khan M Patel SJ Singh I Singh AK. N-Acetyl cysteine protects against injury in a rat model of focal cerebral ischemia. Brain Res. (2003) 971:1–8. 10.1016/S0006-8993(03)02244-3 12691831

[113] Liu Y Liu WC Sun Y Shen X Wang X Shu H. Normobaric hyperoxia extends neuro- and vaso-protection of N-acetylcysteine in transient focal ischemia. Mol Neurobiol. (2017) 54:3418–27. 10.1007/s12035-016-9932-0 27177548

[114] Wishart DS Feunang YD Guo AC Lo EJ Marcu A Grant JR. DrugBank 5.0: a major update to the DrugBank database for 2018. Nucleic Acids Res. (2018) 46:D1074–82. 10.1093/nar/gkx1037 29126136 PMC5753335

[115] Tymianski M. Emerging mechanisms of disrupted cellular signaling in brain ischemia. Nat Neurosci. (2011) 14:1369–73. 10.1038/nn.2951 22030547

[116] Liu Y Wong TP Aarts M Rooyakkers A Liu L Lai TW. NMDA receptor subunits have differential roles in mediating excitotoxic neuronal death both *in vitro* and *in vivo*. J Neurosci. (2007) 27:2846–57. 10.1523/JNEUROSCI.0116-07.2007 17360906 PMC6672582

[117] Petralia RS Wang YX Hua F Yi Z Zhou A Ge L. Organization of NMDA receptors at extrasynaptic locations. Neuroscience. (2010) 167:68–87. 10.1016/j.neuroscience.2010.01.022 20096331 PMC2840201

[118] Hardingham GE. Pro-survival signalling from the NMDA receptor. Biochem Soc Trans. (2006) 34:936–8. 10.1042/BST0340936 17052231 PMC2837915

[119] Zhang SJ Zou M Lu L Lau D Ditzel DA Delucinge-Vivier C. Nuclear calcium signaling controls expression of a large gene pool: identification of a gene program for acquired neuroprotection induced by synaptic activity. PLoS Genet. (2009) 5:e1000604. 10.1371/journal.pgen.1000604 19680447 PMC2718706

[120] Hardingham GE Fukunaga Y Bading H. Extrasynaptic NMDARs oppose synaptic NMDARs by triggering CREB shut-off and cell death pathways. Nat Neurosci. (2002) 5:405–14. 10.1038/nn835 11953750

[121] Ivanov A Pellegrino C Rama S Dumalska I Salyha Y Ben-Ari Y. Opposing role of synaptic and extrasynaptic NMDA receptors in regulation of the extracellular signal-regulated kinases (ERK) activity in cultured rat hippocampal neurons. J Physiol. (2006) 572:789–98. 10.1113/jphysiol.2006.105510 16513670 PMC1779993

[122] Xia P Chen HS Zhang D Lipton SA. Memantine preferentially blocks extrasynaptic over synaptic NMDA receptor currents in hippocampal autapses. J Neurosci. (2010) 30:11246–50. 10.1523/JNEUROSCI.2488-10.2010 20720132 PMC2932667

[123] Joe E Ringman JM. Cognitive symptoms of Alzheimer's disease: clinical management and prevention. BMJ. (2019) 367:l6217. 10.1136/bmj.l6217 31810978

[124] McShane R Westby MJ Roberts E Minakaran N Schneider L Farrimond LE. Memantine for dementia. Cochrane Database Syst Rev. (2019) 3:CD003154. 10.1002/14651858.CD003154.pub6 30891742 PMC6425228

[125] Montagne A Hebert M Jullienne A Lesept F Le Behot A Louessard M. Memantine improves safety of thrombolysis for stroke. Stroke. (2012) 43:2774–81. 10.1161/STROKEAHA.112.669374 22879098

[126] Culmsee C Junker V Kremers W Thal S Plesnila N Krieglstein J. Combination therapy in ischemic stroke: synergistic neuroprotective effects of memantine and clenbuterol. Stroke. (2004) 35:1197–202. 10.1161/01.STR.0000125855.17686.6d 15060319

[127] Trotman M Vermehren P Gibson CL Fern R. The dichotomy of memantine treatment for ischemic stroke: dose-dependent protective and detrimental effects. J Cereb Blood Flow Metab. (2015) 35:230–9. 10.1038/jcbfm.2014.188 25407270 PMC4426739

[128] Tajima N Karakas E Grant T Simorowski N Diaz-Avalos R Grigorieff N. Activation of NMDA receptors and the mechanism of inhibition by ifenprodil. Nature. (2016) 534:63–8. 10.1038/nature17679 27135925 PMC5136294

[129] Zhu S Paoletti P. Allosteric modulators of NMDA receptors: multiple sites and mechanisms. Curr Opin Pharmacol. (2015) 20:14–23. 10.1016/j.coph.2014.10.009 25462287

[130] Christopherson KS Hillier BJ Lim WA Bredt DS. PSD-95 assembles a ternary complex with the N-methyl-D-aspartic acid receptor and a bivalent neuronal NO synthase PDZ domain. J Biol Chem. (1999) 274:27467–73. 10.1074/jbc.274.39.27467 10488080

[131] Sattler R Xiong Z Lu WY Hafner M MacDonald JF Tymianski M. Specific coupling of NMDA receptor activation to nitric oxide neurotoxicity by PSD-95 protein. Science. (1999) 284:1845–8. 10.1126/science.284.5421.1845 10364559

[132] Soriano FX Martel MA Papadia S Vaslin A Baxter P Rickman C. Specific targeting of pro-death NMDA receptor signals with differing reliance on the NR2B PDZ ligand. J Neurosci. (2008) 28:10696–710. 10.1523/JNEUROSCI.1207-08.2008 18923045 PMC2602846

[133] Zong P Feng J Yue Z Li Y Wu G Sun B. Functional coupling of TRPM2 and extrasynaptic NMDARs exacerbates excitotoxicity in ischemic brain injury. Neuron. (2022) 110:1944–58.e8. 10.1016/j.neuron.2022.03.021 35421327 PMC9233078

[134] Nishi T Maier CM Hayashi T Saito A Chan PH. Superoxide dismutase 1 overexpression reduces MCP-1 and MIP-1 alpha expression after transient focal cerebral ischemia. J Cereb Blood Flow Metab. (2005) 25:1312–24. 10.1038/sj.jcbfm.9600124 15829914

[135] Beckman JS. Oxidative damage and tyrosine nitration from peroxynitrite. Chem Res Toxicol. (1996) 9:836–44. 10.1021/tx9501445 8828918

[136] del Zoppo GJ Mabuchi T. Cerebral microvessel responses to focal ischemia. J Cereb Blood Flow Metab. (2003) 23:879–94. 10.1097/01.WCB.0000078322.96027.78 12902832

[137] Squadrito GL Cueto R Splenser AE Valavanidis A Zhang H Uppu RM. Reaction of uric acid with peroxynitrite and implications for the mechanism of neuroprotection by uric acid. Arch Biochem Biophys. (2000) 376:333–7. 10.1006/abbi.2000.1721 10775420

[138] Romanos E Planas AM Amaro S Chamorro A. Uric acid reduces brain damage and improves the benefits of rt-PA in a rat model of thromboembolic stroke. J Cereb Blood Flow Metab. (2007) 27:14–20. 10.1038/sj.jcbfm.9600312 16596120

[139] Onetti Y Dantas AP Perez B Cugota R Chamorro A Planas AM. Middle cerebral artery remodeling following transient brain ischemia is linked to early postischemic hyperemia: a target of uric acid treatment. Am J Physiol Heart Circ Physiol. (2015) 308:H862–74. 10.1152/ajpheart.00001.2015 25637543

[140] Culotta VC Yang M O'Halloran TV. Activation of superoxide dismutases: putting the metal to the pedal. Biochim Biophys Acta. (2006) 1763:747–58. 10.1016/j.bbamcr.2006.05.003 16828895 PMC1633718

[141] Ning Y Huo Y Xue H Du Y Yao Y Sedgwick AC. Tri-Manganese(III) salen-based cryptands: a metal cooperative antioxidant strategy that overcomes ischemic stroke damage *in vivo*. J Am Chem Soc. (2020) 142:10219–27. 10.1021/jacs.0c03805 32390429

[142] Yang K Zeng L Yuan X Wang S Ge A Xu H. The mechanism of ferroptosis regulating oxidative stress in ischemic stroke and the regulation mechanism of natural pharmacological active components. Biomed Pharmacother. (2022) 154:113611. 10.1016/j.biopha.2022.113611 36081288

[143] Alim I Caulfield JT Chen Y Swarup V Geschwind DH Ivanova E. Selenium drives a transcriptional adaptive program to block ferroptosis and treat stroke. Cell. (2019) 177:1262–79.e25. 10.1016/j.cell.2019.03.032 31056284

[144] Ling L Alattar A Tan Z Shah FA Ali T Alshaman R. A potent antioxidant endogenous neurohormone melatonin, rescued MCAO by attenuating oxidative stress-associated neuroinflammation. Front Pharmacol. (2020) 11:1220. 10.3389/fphar.2020.01220 32973495 PMC7472569

[145] Hoye AT Davoren JE Wipf P Fink MP Kagan VE. Targeting mitochondria. Acc Chem Res. (2008) 41:87–97. 10.1021/ar700135m 18193822

[146] Zhou Y Liao J Mei Z Liu X Ge J. Insight into crosstalk between ferroptosis and necroptosis: novel therapeutics in ischemic stroke. Oxid Med Cell Longev. (2021) 2021:9991001. 10.1155/2021/9991001 34257829 PMC8257382

[147] Narang A Qiao F Atkinson C Zhu H Yang X Kulik L. Natural IgM antibodies that bind neoepitopes exposed as a result of spinal cord injury, drive secondary injury by activating complement. J Neuroinflammation. (2017) 14:120. 10.1186/s12974-017-0894-6 28629465 PMC5477255

[148] Elvington A Atkinson C Kulik L Zhu H Yu J Kindy MS. Pathogenic natural antibodies propagate cerebral injury following ischemic stroke in mice. J Immunol. (2012) 188:1460–8. 10.4049/jimmunol.1102132 22198950 PMC3262954

[149] Alawieh A Langley EF Tomlinson S. Targeted complement inhibition salvages stressed neurons and inhibits neuroinflammation after stroke in mice. Sci Transl Med. (2018) 10:eaao6459. 10.1126/scitranslmed.aao6459 29769288 PMC6689196

[150] Alawieh AM Langley EF Feng W Spiotta AM Tomlinson S. Complement-dependent synaptic uptake and cognitive decline after stroke and reperfusion therapy. J Neurosci. (2020) 40:4042–58. 10.1523/JNEUROSCI.2462-19.2020 32291326 PMC7219298

[151] Li Y Lin F. Decoy nanoparticles bearing native C5a receptors as a new approach to inhibit complement-mediated neutrophil activation. Acta Biomater. (2019) 99:330–8. 10.1016/j.actbio.2019.08.033 31446047 PMC7066532

[152] Li J Liu B. The roles and potential therapeutic implications of C5a in the pathogenesis of COVID-19-associated coagulopathy. Cytokine Growth Factor Rev. (2021) 58:75–81. 10.1016/j.cytogfr.2020.12.001 33558131 PMC7733683

[153] Zha H Han X Zhu Y Yang F Li Y Li Q. Blocking C5aR signaling promotes the anti-tumor efficacy of PD-1/PD-L1 blockade. Oncoimmunology. (2017) 6:e1349587. 10.1080/2162402X.2017.1349587 29123963 PMC5665063

[154] Mavroidis M Davos CH Psarras S Varela A C Athanasiadis N Katsimpoulas M. Complement system modulation as a target for treatment of arrhythmogenic cardiomyopathy. Basic Res Cardiol. (2015) 110:27. 10.1007/s00395-015-0485-6 25851234

[155] Xu GL Chen J Yang F Li GQ Zheng LX Wu YZ. C5a/C5aR pathway is essential for the pathogenesis of murine viral fulminant hepatitis by way of potentiating Fgl2/fibroleukin expression. Hepatology. (2014) 60:114–24. 10.1002/hep.27114 24604562

[156] Orsini F Villa P Parrella S Zangari R Zanier ER Gesuete R. Targeting mannose-binding lectin confers long-lasting protection with a surprisingly wide therapeutic window in cerebral ischemia. Circulation. (2012) 126:1484–94. 10.1161/CIRCULATIONAHA.112.103051 22879370 PMC3478764

[157] Mercurio D Piotti A Valente A Oggioni M Ponstein Y Van Amersfoort E. Plasma-derived and recombinant C1 esterase inhibitor: binding profiles and neuroprotective properties in brain ischemia/reperfusion injury. Brain Behav Immun. (2021) 93:299–311. 10.1016/j.bbi.2021.01.002 33444732

[158] Liesz A Zhou W Mracsko E Karcher S Bauer H Schwarting S. Inhibition of lymphocyte trafficking shields the brain against deleterious neuroinflammation after stroke. Brain. (2011) 134:704–20. 10.1093/brain/awr008 21354973

[159] Lu Y Zhou M Li Y Li Y Hua Y Fan Y. Minocycline promotes functional recovery in ischemic stroke by modulating microglia polarization through STAT1/STAT6 pathways. Biochem Pharmacol. (2021) 186:114464. 10.1016/j.bcp.2021.114464 33577892

[160] Abdul Y Abdelsaid M Li W Webb RC Sullivan JC Dong G. Inhibition of toll-like receptor-4 (TLR-4) improves neurobehavioral outcomes after acute ischemic stroke in diabetic rats: possible role of vascular endothelial TLR-4. Mol Neurobiol. (2019) 56:1607–17. 10.1007/s12035-018-1184-8 29909454 PMC6295357

[161] Wang Y Ge P Zhu Y. TLR2 and TLR4 in the brain injury caused by cerebral ischemia and reperfusion. Mediators Inflamm. (2013) 2013:124614. 10.1155/2013/124614 23864765 PMC3706022

[162] Zhai Y Zhu Y Liu J Xie K Yu J Yu L. Dexmedetomidine post-conditioning alleviates cerebral ischemia-reperfusion injury in rats by inhibiting high mobility group protein B1 group (HMGB1)/toll-like receptor 4 (TLR4)/nuclear factor kappa B (NF-kappaB) Signaling Pathway. Med Sci Monit. (2020) 26:e918617. 10.12659/MSM.918617 31912804 PMC6977611

[163] Chen Y Brennan-Minnella AM Sheth S El-Benna J Swanson RA. Tat-NR2B9c prevents excitotoxic neuronal superoxide production. J Cereb Blood Flow Metab. (2015) 35:739–42. 10.1038/jcbfm.2015.16 25669908 PMC4420863

[164] Zhou L Li F Xu HB Luo CX Wu HY Zhu MM. Treatment of cerebral ischemia by disrupting ischemia-induced interaction of nNOS with PSD-95. Nat Med. (2010) 16:1439–43. 10.1038/nm.2245 21102461

[165] Lai TW Zhang S Wang YT. Excitotoxicity and stroke: identifying novel targets for neuroprotection. Prog Neurobiol. (2014) 115:157–88. 10.1016/j.pneurobio.2013.11.006 24361499

[166] Xiong Z Chang L Qu Y Pu Y Wang S Fujita Y. Neuronal brain injury after cerebral ischemic stroke is ameliorated after subsequent administration of (R)-ketamine, but not (S)-ketamine. Pharmacol Biochem Behav. (2020) 191:172904. 10.1016/j.pbb.2020.172904 32156500

[167] Huang B Chen P Huang L Li S Zhu R Sheng T. Geniposide attenuates post-ischaemic neurovascular damage *via* GluN2A/AKT/ERK-dependent mechanism. Cell Physiol Biochem. (2017) 43:705–16. 10.1159/000480657 28957809

[168] Liu Y Fu X Liu Y Zhang T Cui P Wang S. Neuroprotective effect of pseudoginsenoside-F11 on permanent cerebral ischemia in rats by regulating calpain activity and NR2A submit-mediated AKT-CREB signaling pathways. Phytomedicine. (2022) 96:153847. 10.1016/j.phymed.2021.153847 34836744

[169] Wang XX Wang F Mao GH Wu JC Li M Han R. NADPH is superior to NADH or edaravone in ameliorating metabolic disturbance and brain injury in ischemic stroke. Acta Pharmacol Sin. (2022) 43:529–40. 10.1038/s41401-021-00705-5 34168317 PMC8888674

[170] Haberman F Tang SC Arumugam TV Hyun DH Yu QS Cutler RG. Soluble neuroprotective antioxidant uric acid analogs ameliorate ischemic brain injury in mice. Neuromolecular Med. (2007) 9:315–23. 10.1007/s12017-007-8010-1 17999205

[171] Zhi SM Fang GX Xie XM Liu LH Yan J Liu DB. Melatonin reduces OGD/R-induced neuron injury by regulating redox/inflammation/apoptosis signaling. Eur Rev Med Pharmacol Sci. (2020) 24:1524–36. 32096202 10.26355/eurrev_202002_20211

[172] Hou Y Wang Y He Q Li L Xie H Zhao Y. Nrf2 inhibits NLRP3 inflammasome activation through regulating Trx1/TXNIP complex in cerebral ischemia reperfusion injury. Behav Brain Res. (2018) 336:32–9. 10.1016/j.bbr.2017.06.027 28851669

[173] Chang G Chen Y Zhang H Zhou W. Trans sodium crocetinate alleviates ischemia/reperfusion-induced myocardial oxidative stress and apoptosis *via* the SIRT3/FOXO3a/SOD2 signaling pathway. Int Immunopharmacol. (2019) 71:361–71. 10.1016/j.intimp.2019.03.056 30952100

[174] Zhao B Sun LK Jiang X Zhang Y Kang J Meng H. Genipin protects against cerebral ischemia-reperfusion injury by regulating the UCP2-SIRT3 signaling pathway. Eur J Pharmacol. (2019) 845:56–64. 10.1016/j.ejphar.2018.12.028 30582911

[175] De Ceunynck K De Meyer SF Vanhoorelbeke K. Unwinding the von Willebrand factor strings puzzle. Blood. (2013) 121:270–7. 10.1182/blood-2012-07-442285 23093621

[176] De Meyer SF Denorme F Langhauser F Geuss E Fluri F Kleinschnitz C. Thromboinflammation in stroke brain damage. Stroke. (2016) 47:1165–72. 10.1161/STROKEAHA.115.011238 26786115

[177] Sonneveld MA de Maat MP Portegies ML Kavousi M Hofman A Turecek PL. Low ADAMTS13 activity is associated with an increased risk of ischemic stroke. Blood. (2015) 126:2739–46. 10.1182/blood-2015-05-643338 26511134

[178] Kim SJ Jenne CN. Role of platelets in neutrophil extracellular trap (NET) production and tissue injury. Semin Immunol. (2016) 28:546–54. 10.1016/j.smim.2016.10.013 27876233

[179] Laridan E Denorme F Desender L Francois O Andersson T Deckmyn H. Neutrophil extracellular traps in ischemic stroke thrombi. Ann Neurol. (2017) 82:223–32. 10.1002/ana.24993 28696508

[180] Amaro S Laredo C Renu A Llull L Rudilosso S Obach V. Uric acid therapy prevents early ischemic stroke progression: a tertiary analysis of the URICO-ICTUS trial (efficacy study of combined treatment with uric acid and r-tPA in acute ischemic stroke). Stroke. (2016) 47:2874–6. 10.1161/STROKEAHA.116.014672 27758945

[181] Pardridge WM. Blood-brain barrier drug targeting: the future of brain drug development. Mol Interv. (2003) 3:90–105.51. 10.1124/mi.3.2.90 14993430

[182] D'Souza A Dave KM Stetler RA S Manickam D. Targeting the blood-brain barrier for the delivery of stroke therapies. Adv Drug Deliv Rev. (2021) 171:332–51. 10.1016/j.addr.2021.01.015 33497734

[183] Patel T Zhou J Piepmeier JM Saltzman WM. Polymeric nanoparticles for drug delivery to the central nervous system. Adv Drug Deliv Rev. (2012) 64:701–5. 10.1016/j.addr.2011.12.006 22210134 PMC3323692

[184] He W Zhang Z Sha X. Nanoparticles-mediated emerging approaches for effective treatment of ischemic stroke. Biomaterials. (2021) 277:121111. 10.1016/j.biomaterials.2021.121111 34488117

[185] Gabathuler R. Approaches to transport therapeutic drugs across the blood-brain barrier to treat brain diseases. Neurobiol Dis. (2010) 37:48–57. 10.1016/j.nbd.2009.07.028 19664710

[186] Wohlfart S Gelperina S Kreuter J. Transport of drugs across the blood-brain barrier by nanoparticles. J Control Release. (2012) 161:264–73. 10.1016/j.jconrel.2011.08.017 21872624

[187] Rak J. Extracellular vesicles - biomarkers and effectors of the cellular interactome in cancer. Front Pharmacol. (2013) 4:21. 10.3389/fphar.2013.00021 23508692 PMC3589665

[188] Lener T Gimona M Aigner L Borger V Buzas E Camussi G. Applying extracellular vesicles based therapeutics in clinical trials - an ISEV position paper. J Extracell Vesicles. (2015) 4:30087. 10.3402/jev.v4.30087 26725829 PMC4698466

[189] Zhang ZG Buller B Chopp M. Exosomes - beyond stem cells for restorative therapy in stroke and neurological injury. Nat Rev Neurol. (2019) 15:193–203. 10.1038/s41582-018-0126-4 30700824

[190] Hwang DW Choi H Jang SC Yoo MY Park JY Choi NE. Noninvasive imaging of radiolabeled exosome-mimetic nanovesicle using (99m)Tc-HMPAO. Sci Rep. (2015) 5:15636. 10.1038/srep15636 26497063 PMC4620485

[191] Webb RL Kaiser EE Scoville SL Thompson TA Fatima S Pandya C. Human neural stem cell extracellular vesicles improve tissue and functional recovery in the murine thromboembolic stroke model. Transl Stroke Res. (2018) 9:530–9. 10.1007/s12975-017-0599-2 29285679 PMC6132936

[192] Terstappen GC Meyer AH Bell RD Zhang W. Strategies for delivering therapeutics across the blood-brain barrier. Nat Rev Drug Discov. (2021) 20:362–83. 10.1038/s41573-021-00139-y 33649582

[193] Hossmann KA. The two pathophysiologies of focal brain ischemia: implications for translational stroke research. J Cereb Blood Flow Metab. (2012) 32:1310–6. 10.1038/jcbfm.2011.186 22234335 PMC3390813

[194] Mastellos DC Ricklin D Lambris JD. Clinical promise of next-generation complement therapeutics. Nat Rev Drug Discov. (2019) 18:707–29. 10.1038/s41573-019-0031-6 31324874 PMC7340853

[195] Chong WP Mattapallil MJ Raychaudhuri K Bing SJ Wu S Zhong Y. The cytokine IL-17A limits Th17 pathogenicity *via* a negative feedback loop driven by autocrine induction of IL-24. Immunity. (2020) 53:384–97.e5. 10.1016/j.immuni.2020.06.022 32673565 PMC7362799

[196] Lee JY Hall JA Kroehling L Wu L Najar T Nguyen HH. Serum amyloid A proteins induce pathogenic Th17 cells and promote inflammatory disease. Cell. (2020) 183:2036–9. 10.1016/j.cell.2020.12.008 33357400 PMC7891798

